# Discovery of disease-associated cellular states using ResidPCA in single-cell RNA and ATAC sequencing data

**DOI:** 10.1016/j.xhgg.2025.100538

**Published:** 2025-10-27

**Authors:** Shaye Carver, Kodi Taraszka, Stefan Groha, Alexander Gusev

**Affiliations:** 1 Program in Biological and Biomedical Sciences, Harvard Medical School, Boston, MA, USA; 2 Division of Population Sciences, Dana-Farber Cancer Institute and Harvard Medical School, Boston, MA, USA; 3 Division of Genetics, Brigham & Women’s Hospital, Boston, MA, USA; 4 The Broad Institute, Cambridge, MA, USA

**Keywords:** single-cell sequencing, cellular heterogeneity, disease-associated states, scRNA-seq, snATAC-seq, Alzheimer disease, cell state identification, principal-component analysis

## Abstract

To advance understanding of cellular heterogeneity in disease from single-cell sequencing data, we introduce residual principal-component analysis (ResidPCA), a robust method for identifying cell states that explicitly models cell-type heterogeneity. In simulations, ResidPCA achieved more than 4-fold higher accuracy than conventional PCA and over 3-fold higher accuracy than non-negative matrix factorization (NMF)-based methods in detecting states expressed across multiple cell types. Applied to single-cell RNA sequencing of light-stimulated mouse visual cortex cells, ResidPCA captured stimulus-driven variability with an accuracy more than 5-fold higher than NMF-based approaches. In single-nucleus datasets from an Alzheimer disease cohort, ResidPCA identified 44 chromatin accessibility-based states from single-nucleus ATAC-seq (snATAC-seq) and 42 transcriptional states from single-nucleus RNA-seq. Thirty snATAC-seq states were significantly enriched for Alzheimer disease heritability, often more so than established cell types such as microglia. The snATAC-seq state most significantly enriched for heritability further elucidates a recently implicated neuron-oligodendrocyte-microglial mechanistic axis, linking early amyloid production in neurons and oligodendrocytes with later microglial activation and immune response. These results highlight the ability of ResidPCA to uncover previously hidden biological variation in single-cell data and reveal disease-relevant cell states.

## Teaser

Residual principal-component analysis (ResidPCA) identifies disease-relevant cell states by modeling heterogeneity across cell types. Applied to mouse visual cortex and Alzheimer disease datasets, ResidPCA outperformed existing methods and uncovered chromatin and transcriptional states enriched for heritability, offering mechanistic insights into neurodegeneration.

## Introduction

Single-cell omic measurements have been used to dissect the heterogeneity of cell populations at unprecedented resolution, with a focus on cell-type inference.^1^^,^^2^ The integration of genome-wide association studies (GWASs) with omics data has shown that many diseases, including neurodegenerative disorders, are linked to and even modulate disease through a diverse range of cell types.^3^^,^^4^^,^^5^^,^^6^^,^^7^^,^^8^ Despite their disease relevance, only a small fraction of heritability is mediated by existing gene expression measurements due to the complexity of regulatory effects across tissues and cell types.^6^ Recent studies have attempted to implicate the missing heritability by moving beyond canonical cell types to more granular cell “states,” which capture the underlying functional mechanisms of tissues and cell types.^5^^,^^9^^,^^10^^,^^11^^,^^12^^,^^13^^,^^14^ These causal mechanisms provide deeper insights into the biological changes driving disease phenotypes and aid in identifying more precise druggable targets compared to a broad cell-type approach.^14^

Previous efforts in identifying cell states have relied largely on methodologies borrowed from cell-type inference. These approaches can lack sensitivity to both identify all active states within a dataset and connect them to specific traits or diseases. For instance, many studies adopt unsupervised clustering methods to identify cell states initially designed for discrete cell-type identification.^7^^,^^15^^,^^16^ This strategy may erroneously partition continuous cell states into discrete clusters, restricting each cell to a singular state—a biologically plausible approach for identifying cell types but not states, where cells are capable of occupying multiple states concurrently.^11^ This approach likewise restricts the exploration of cellular states to those within a specific cell type, thus limiting the discovery of potential shared states that span multiple cell types.

To address these limitations, methods have been introduced for inferring continuous cell representations using various dimensionality-reduction implementations. Such methods include non-negative matrix factorization (NMF),^17^ consensus NMF (cNMF),^12^ principal-component analysis (PCA),^14^^,^^18^ uniform manifold approximation and projection (UMAP) with unsupervised clustering,^15^^,^^16^^,^^19^^,^^20^^,^^21^ and co-expression analyses.^22^ However, no existing methods model cell-type heterogeneity—the effect of cell type on expression—to accurately estimate cell states that are independent of cell type. Additionally, the sensitivity of existing methods can be severely limited by cell-type heterogeneity, which is the largest source of biological variation in single-cell datasets.^23^^,^^24^ While some techniques attempt to address cell-type-driven noise by applying PCA to individual cell types (which we refer to as iterative PCA), this compromises statistical power by reducing sample size and may miss states that are rare or present in multiple cell types.^14^

Here, we develop and benchmark our method, residual PCA (ResidPCA). This method addresses cell-type heterogeneity by utilizing known cell-type labels to efficiently denoise single-cell datasets, resulting in the elimination of irrelevant and noisy biological signals that can obscure accurate cell-state identification. Through analyses of simulated and real data, we demonstrate that ResidPCA exhibits higher sensitivity than existing approaches, particularly in scenarios featuring many cell types, states spanning multiple cell types, or rare cell states. In an application of ResidPCA to human brain-derived single-nuclei RNA sequencing (snRNA-seq) and single-nuclei assay for transposase accessible chromatin sequencing (snATAC-seq) data, we identify 86 total states accounting for a higher fraction of Alzheimer disease (AD) heritability compared to previously established cell-type enrichments and map states to their respective biologically relevant pathways. We introduce an efficient implementation of ResidPCA that is 3.5 times faster and 5 times more resource efficient than current state-of-the-art methods.

## Material and methods

### Generative model for cellular states in single-cell data

Let $Z\in {\mathbb{R}}^{I\times J}$ represent the log-normalized expression matrix, where I denotes the total number of cells and J denotes the total number of genes measured in a single-cell experiment. Each entry $z_{ij}$ in this matrix corresponds to the log-normalized reads per 10,000 (transcripts per 10,000 [TP10K]) for gene j within cell i. This normalization accounts for differences in sequencing depth across cells, providing a standardized measure of gene expression.

To model $z_{ij}$, we parameterize it using a Gaussian distribution, which captures the underlying variability and structure in the expression data:

$$z_{ij}∼N\;\left(\theta_{ij}^{\mathrm{CT}}+\theta_{ij}^{\mathrm{ST}},\;\sigma_{j}^{2}\right)\text{,}$$

where the mean of $z_{ij}$ is the sum of 2 components: the cell-type mean expression, $\theta_{ij}^{\mathrm{CT}}$, and the cell-state mean expression, $\theta_{ij}^{\mathrm{ST}}$. Let $\sigma_{j}^{2}$ denote the variance for gene j.

Next, we define $Y\in {\mathbb{R}}^{I\times J}$ as the observed raw count matrix, which is a transformation of Z. In this matrix, each entry $y_{ij}$ represents the actual number of reads (or counts) for gene j in cell i. These raw counts are the direct output from sequencing, capturing the number of transcripts in scRNA-seq or the number of peaks in scATAC-seq data.

To model $y_{ij}$, we parameterize it using a gamma-Poisson distribution with respect to the cell-type mean expression ($\theta_{ij}^{\mathrm{CT}}$) and cell-state mean expression ($\theta_{ij}^{\mathrm{ST}}$):

$$y_{ij}∼\mathrm{GammaPoisson}\;\left(n_{i}\;\exp\;\left(\theta_{ij}^{\mathrm{CT}}+\theta_{ij}^{\mathrm{ST}}\right),\;\phi_{j}\right)\text{,}$$

where $n_{i}$ represents a size factor, such as the total number of unique molecular identifiers per cell, and $\phi_{j}$ is the overdispersion of gene j. The gamma-Poisson distribution is commonly employed to model raw expression data because it captures data variability without introducing unnecessary complexity into the statistical model.^25^

### Connecting ResidPCA to single-cell generative model

We now introduce our method ResidPCA, which leverages log-normalized TP10K data ($z_{ij}$) with known cell-type labels to estimate the contributions of both cell type and cell state on expression.

The cell-type component ($\theta_{ij}^{\mathrm{CT}}$) can be expressed as the following inner product:

$$\theta_{ij}^{\mathrm{CT}}=\langle U_{i,:}^{\mathrm{CT}},V_{j}^{\mathrm{CT}}\rangle \text{,}$$

where $U_{i,:}^{\mathrm{CT}}$ is the cell-type indicator vector for observed cell i and is the i-th row of the cell-type indicator matrix $U^{\mathrm{CT}}\in {\mathbb{R}}^{I\times M}$, where I is the number of cells and M is the number of distinct cell types. Additionally, $V_{j}^{\text{CT}}$ is the normalized mean cell-type expression for gene j and is the j-th column of the cell-type mean expression matrix $V^{\mathrm{CT}}\in {\mathbb{R}}^{M\times J}$, where J is the number of genes.

Similarly, the state-driven component ($\theta_{ij}^{\mathrm{ST}}$) is decomposed into the inner product of the state-driven cell embeddings or PCs and the state-driven gene loadings represented below:

$$\theta_{ij}^{\mathrm{ST}}=\langle U_{i,:}^{\mathrm{ST}},V_{j}^{\mathrm{ST}}\rangle \text{,}$$

where $U_{i,:}^{\mathrm{ST}}$ is the i-th row of the cell-state matrix $U^{\mathrm{ST}}\in {\mathbb{R}}^{I\times L}$, which represents how active each of the I cells are in a set of cell states L. Separately, $V_{j}^{\mathrm{ST}}$ is the j-th column of the cell-state mean expression matrix $V^{\mathrm{ST}}\in {\mathbb{R}}^{L\times J}$, representing how active the set of genes J is across a set of L states.

Given the above decomposition of $\theta_{ij}^{\mathrm{CT}}$ and $\theta_{ij}^{\text{ST}}$, the normalized TP10K expression data ($z_{ij}$) can be redefined as:

$$z_{ij}∼N\;\left(\langle U_{i,:}^{\mathrm{CT}},V_{j}^{\mathrm{CT}}\rangle +\langle U_{i,:}^{\mathrm{ST}},V_{j}^{\mathrm{ST}}\rangle ,\;\sigma_{j}^{2}\right)\text{.}$$

We note that the raw count data $y_{i}j$ can also be redefined as a function of $U^{\text{ST}}$, $V^{\text{ST}}$, $U^{\text{CT}}$, and $V^{\text{CT}}$.

ResidPCA learns the underlying parameters of $Z\in {\mathbb{R}}^{I\times J}$ across all genes and cells, namely $V^{\text{CT}}$, $U^{\text{ST}}$, and $V^{\text{ST}}$, using a 2-step procedure. In the first step, $V^{\text{CT}}$ is learned through linear regression, as $U^{\text{CT}}$ and Z are known *a priori*, by minimizing the following negative log likelihood (i.e., computing the residual):

$$\min_{V_{j}^{\mathrm{CT}}}\sum_{i}{\left(z_{ij}-\langle U_{i,:}^{\mathrm{CT}},V_{j}^{\mathrm{CT}}\rangle \right)}^{2}\text{.}$$

Once $V^{\text{CT}}$ is learned, maximizing the likelihood of the Gaussian distribution describing $z_{ij}$ is equivalent to minimizing the following objective function across cells in the second step of our procedure:

$$\min_{U_{i}^{ST},\;V_{j}^{ST}}\sum_{i}{\left[z_{ij}-\left(\langle U_{i,:}^{CT},V_{j}^{CT}\rangle +\langle U_{i,:}^{ST},V_{j}^{ST}\rangle \right)\right]}^{2}\text{.}$$

When the above minimization is subject to the orthogonality constraint,

$$V_{j}^{{ST}^{\prime }}V_{k}^{ST}=0\;\text{for}\;j\neq k\text{.}$$

the solution becomes equivalent to using PCA to learn the normalized reconstruction of state-driven expression by minimizing the reconstruction error (or Euclidean distance) between the input data Z and a low-rank, orthogonal representation of the data. The number of states learned, L, is a hyperparameter, and its inference is discussed below (see computing the number of significant components/latent features).

### ResidPCA inference model

The goal of ResidPCA is to infer cell states that are independent of cell type. The 2 state-related parameters inferred by ResidPCA are the normalized cell embeddings ($U^{\text{ST}}$) and the normalized gene loadings ($V^{\text{ST}}$) for a set of L states. The state-related cell embeddings quantify the degree to which each cell is involved in a particular state, while the state-related gene loadings measure the contribution of each gene to the set of states. Additionally, ResidPCA infers a cell-type-related parameter in an intermediate step: the normalized cell-type gene loadings ($V^{\text{CT}}$), which capture the average expression effect of each gene j across the cell types, or the average expression profile of each cell type.

ResidPCA requires 2 inputs. The first input is the cell-type labels, represented by an indicator or probability matrix, $U^{\text{CT}}$. We note that $U^{\text{CT}}$ can also be a probability matrix to account for uncertainty in cell-type labels and that other covariates can also be included in the matrix. The second input is the normalized TP10K log-transformed expression matrix, $Z\in {\mathbb{R}}^{I\times J}$. This normalization process accounts for sequencing depth, stabilizes variance to reduce skewness, and minimizes the impact of outliers. As a result, the distribution of gene expression data becomes approximately Gaussian, with a linear mean-variance relationship. This transformation aligns the data with the assumptions of PCA, including homoscedasticity, linearity, normality, and mean centering.

ResidPCA is performed in the following steps.

Step 1. Cell-type label acquisition. The cell type for each input cell, indexed by i=1,…,I, must be inferred. This can be accomplished using established methodologies such as clustering techniques,^26^ marker gene analysis, or integration^27^ with reference datasets. These labels must be obtained prior to performing ResidPCA and are used as input to the analysis.
Step 2. Estimation of cell-type-specific expression component. For each gene j, the cell-type-specific expression component ${\hat{Z}}_{j}^{\text{CT}}$ is estimated via linear regression. This regression models the relationship between the cell-type indicator (or probability matrix, if applicable) $U^{\text{CT}}$ and the total normalized expression Z. The regression coefficients $V^{\text{CT}}$ represent the mean expression of gene j across different cell types:

$$Z_{j}∼U^{\mathrm{CT}}V_{j}^{\mathrm{CT}}\text{.}$$

To compute the cell-type-specific expression component ${\hat{Z}}_{j}^{\mathrm{CT}}$ for a given gene j, we derive the reconstruction estimates from the regression:

$${\hat{Z}}_{j}^{\mathrm{CT}}=U^{\mathrm{CT}}{\hat{V}}_{j}^{\mathrm{CT}},$$

where ${\hat{V}}_{j}^{\mathrm{CT}}$ represents the estimated regression coefficients or betas outputted by the regression for a given gene j. This step enables precise estimation of gene expression attributable to specific cell types, facilitating the further analysis of state-driven expression components.

Note that instead of computing ${\hat{Z}}_{j}^{\mathrm{CT}}$ gene by gene, as done in many single-cell analysis tools such as Seurat,^28^ we compute the matrix ${\hat{Z}}_{}^{\mathrm{CT}}$ for all genes simultaneously, offering significantly faster performance compared to other implementations.

Step 3. Estimation of state-driven expression component. To estimate the state-driven expression component ${\hat{Z}}_{j}^{\mathrm{ST}}$, we first subtract the estimated cell-type-driven expression component ${\hat{Z}}_{j}^{\mathrm{CT}}$ from the total TP10K-normalized expression $Z_{j}$. This yields an expression component that is independent of cell type and represents the additive state-driven expression component of gene j across all states:

$${\hat{Z}}_{j}^{\mathrm{ST}}=Z_{j}-U^{\mathrm{CT}}{\hat{V}}_{j}^{\mathrm{CT}}=Z_{j}-{\hat{Z}}_{j}^{\text{CT}}\text{.}$$

Note that the estimator ${\hat{Z}}_{j}^{\text{ST}}$ is unbiased with respect to state-driven expression that is independent of cell type. However, it becomes biased if the state-driven expression is dependent or correlated with 1 or more cell types. This bias arises because any state-driven expression correlated with cell type is regressed out of the total expression matrix.

Step 4. Decomposition of state-driven expression component. To decompose the estimated total state-driven expression component ${\hat{Z}}_{j}^{\text{ST}}$, across all states into distinct states, we apply PCA to the standardized covariance matrix of the estimated state-driven expression component across all genes. Using PCA, which is equivalent to the singular value decomposition (SVD) applied to ${\hat{Z}}_{j}^{\text{ST}}$, we identify the state-driven cell embeddings, $U^{\text{ST}}$, across all cells, the state-related gene loadings, $V^{\text{ST}}$, across all genes, and the corresponding eigenvalues:

$${\hat{Z}}^{\mathrm{ST}}={\hat{U}}^{\mathrm{ST}}\Lambda^{\mathrm{ST}}{\hat{V}}^{\mathrm{ST}}\;\text{(SVD).}$$

Here, ${\hat{U}}^{\mathrm{ST}}\in {\mathbb{R}}^{I\times L}$ represents the matrix of PCs, illustrating the continuum on which each cell expresses each state. The diagonal matrix $\Lambda^{\mathrm{ST}}\in {\mathbb{R}}^{L\times L}$ contains singular eigenvalues, ordered by descending variance explained or importance of each PC. The matrix ${\hat{V}}^{\mathrm{ST}}\in {\mathbb{R}}^{L\times J}$ denotes the gene loadings matrix or eigenvectors, indicating the extent to which each gene is expressed within a given state. (The effects of cell type and cell state on expression on $\hat{Z}$ are assumed to be linear.).

### Simulation: Synthetic gene expression dataset generation

We generated synthetic gene expression datasets incorporating a set of cell types and a one (single) cell state for simplicity. This was achieved by extending the simulation framework introduced by GlmGamPoi^29^ to simulate concurrent cell-state-driven expression alongside cell-type-specific expression. Initially, we fit GlmGamPoi to the expression profiles of each cell type separately, using the snRNA-seq data from Morabito et al.^15^ This allowed us to estimate both the mean cell-type expression count vector, ${\hat{\mu }}_{j}^{\mathrm{CT}}$, and the overdispersion vector, ${\hat{\Phi }}_{j}^{\mathrm{CT}}$, for each gene across the 7 cell types in the dataset. Metrics were estimated for 2,527 genes, comprising the union of the top 500 most variable genes in each cell type.

The mean state-driven expression count vector $\mu_{j}^{\mathrm{ST}}$ for all cell types was defined as a function of the mean cell-type expression count, ${\hat{\mu }}_{j}^{\mathrm{CT}}$, where:

$${\hat{\mu }}_{j}^{\mathrm{ST}}={\text{FC}}_{j}^{\mathrm{ST}}\;{\hat{\mu }}_{j}^{\mathrm{CT}}$$

Here, ${\text{FC}}_{j}^{\mathrm{ST}}$ represents the fold change in gene j relative to the baseline mean gene expression for each cell type, ${\hat{\mu }}_{j}^{\mathrm{CT}}$. The fold change, $FC_{j}^{\text{ST}}$, is sampled from:

$${\text{FC}}_{j}^{\mathrm{ST}}∼U([0.125,0.707],[1.414,8])$$

ensuring that

$$\log\;FC_{j}^{\text{ST}}∼U\left(\left[-3,-0.5\right],\left[0.5,3\right]\right)\text{.}$$

This is consistent with the realistic logFC of differentially expressed genes in sequencing data. The transformation reflects that mean cell-state-based gene expression is a fold change difference from mean cell-type-based expression.

The mean total expression count for a given cell i and gene j is defined as:

$$\mu_{ij}^{\mathrm{CT}+\mathrm{ST}}=\frac{{\left(\omega_{i}^{\mathrm{CT}}\right)}^{'}{\hat{\mu }}_{j}^{\mathrm{CT}}+\omega_{i}^{\mathrm{ST}}{\left(\omega_{i}^{\mathrm{CT}}\right)}^{'}{\hat{\mu }}_{j}^{\mathrm{ST}}C_{i}^{\mathrm{ST}}G_{j}^{\mathrm{ST}}}{\|\omega_{i}^{\mathrm{CT}}{\|}_{1}+\omega_{i}^{\mathrm{ST}}C_{i}^{\mathrm{ST}}G_{j}^{\mathrm{ST}}},$$

where $\omega_{i}^{\mathrm{CT}}$ is the indicator vector for the identity of a cell, and $\omega_{i}^{\mathrm{ST}}$ is a scalar representing the continuum on which cell i expresses the state, following a standard uniform distribution $\omega_{i}^{\mathrm{ST}}∼U(0,1)$. $C_{i}^{\mathrm{ST}}$ is a binary indicator for state expression in cell i, and $G_{j}^{\text{ST}}$ is a binary indicator for state expression in gene j.

After generating $\mu_{ij}^{CT+ST}$, the mean total expression capturing both cell type and state-driven expression, an expression count for gene j in cell i is sampled from:

$$y_{ij}^{\mathrm{CT}+\mathrm{ST}}∼\mathrm{GammaPoisson}\;\left(\mu_{ij}^{\mathrm{CT}+\mathrm{ST}},{\left(\omega_{i}^{\mathrm{CT}}\right)}^{'}{\hat{\Phi }}_{j}^{\mathrm{CT}}\right).$$

Simulations were conducted to generate count-based gene expression matrices reflecting varying levels of cell-type heterogeneity. Datasets included 7 cell types to represent low heterogeneity, matching the Morabito et al. dataset,^15^ and 100 cell types to represent high heterogeneity. For scenarios with 7 cell types, 20,000 cells were simulated, and with 100 cell types, 40,000 cells were simulated. In the latter scenario, the 7 fitted cell type parameters from the Morabito dataset^15^ were used alongside 93 *in silico*-generated cell-type parameters. Mean and overdispersion parameters for these additional cell types were generated by sampling from a uniform distribution bounded by the minimum and maximum parameters of ${\hat{\mu }}_{j}^{\mathrm{CT}}$ and ${\hat{\Phi }}_{j}^{\mathrm{CT}}$ from the real data:

$$\mu_{j}^{CT_{silico}}∼U\;\left(\min({\hat{\mu }}_{j}^{\mathrm{CT}}),\max({\hat{\mu }}_{j}^{\mathrm{CT}})\right)$$

and

$$\Phi_{j}^{{\mathrm{CT}}_{\mathrm{silico}}}∼U\;\left(\min({\hat{\Phi }}_{j}^{\mathrm{CT}}),\max({\hat{\Phi }}_{j}^{\mathrm{CT}})\right).$$

Expression matrices were generated to include states within a single cell type or across all cell types, with states ranging from rare to abundant. This was controlled by varying the number of genes and cells expressing the state, ranging from 0.15 to 0.7 (0.15, 0.3, 0.5, 0.7) of the total genes and cells capable of expressing the state. Cells and genes involved in a given state were randomly sampled, and simulations for each parameter setting were repeated with 10 different seeds.

The simulated datasets were consistent with real snRNA-seq and gene-mapped snATAC-seq datasets in terms of sparsity, mean-variance relationship, and UMAP embedding distribution.

### Computing the number of significant components/latent features

To determine the number of significant components or states recovered by PCA, we used the Bayesian information criterion (BIC) method adapted for PCA.^30^ The BIC is a statistical criterion used to select the best model by balancing model fit and complexity, penalizing models with more parameters to avoid overfitting. This method is a function of the total number of cells, denoted as I, the total number of genes, denoted as J, and the sample eigenvalues, λ, obtained from the diagonals of the eigenvalue matrix, Λ. The optimal number of PCs to include in the model is estimated by first computing the BIC value, denoted as ${\text{BIC}}_{p}$. Each model p includes the first p PCs, where p∈0,1,…,J-1. The dimensionality of the data, or the number of significant PCs to retain as significant, is determined by identifying the value of p that corresponds to the PC minimizing the BIC value, expressed as $\arg\;\min_{p}{\mathrm{BIC}}_{p}$. The BIC value is computed as follows:

$${\mathrm{BIC}}_{p}=I\log\;\left(\prod_{k=1}^{p}\lambda_{k}\right)+I(J-p)\log\;\left(\frac{1}{J-p}\sum_{t=p+1}^{J}\lambda_{t}\right)+(\log I)d_{j}+C_{I,J}\text{,}$$

where

$$C_{I,J}=I{\left[\log\;\left(\frac{I-1}{I}\right)\right]}^{J}+IJ\left\{1+\log\;\left(2\pi \right)\right\}$$

and

$$d_{p}=(p+1)\;\left(J+1-\frac{p}{2}\right)$$

capture the number of independent parameters in the model.

The computation of the sum of eigenvalues greater than p, $\sum_{t=p+1}^{J}\lambda_{t}$, requires evaluating all J eigenvalues, where J may be as large as 20,000, corresponding to 20,000 total genes. However, to reduce computational and memory demands, only the first 200 eigenvalues or BIC cutoffs were computed, as the significance of PCs was found to be negligible beyond this point. This approach minimizes the computational burden of PCA and the number of BIC cutoff calculations. To avoid computing all eigenvalues, we utilize the fact that the sum of the eigenvalues is equivalent to the trace of the standardized covariance matrix:

$$\sum_{t=1}^{J}\lambda_{t}=\text{tr}\left(\frac{1}{I-1}\sum_{l=1}^{I}\left(z_{i}-\bar{z}\right){\left(z_{i}-\bar{z}\right)}^{T}\right)\text{,}$$

where $\bar{z}=\frac{1}{I}\sum_{l=1}^{I}z_{l}$ is the average expression across samples. Therefore, the sum of the eigenvalues from p+1 to J is given by:

$$\sum_{t=p+1}^{J}\lambda_{t}=\text{tr}\left(\frac{1}{I-1}\sum_{l=1}^{I}\left(z_{I}-\bar{z}\right){\left(z_{I}-\bar{z}\right)}^{T}\right)-\sum_{t=1}^{p}\lambda_{t}$$

For PCA-based methods, the elbow plot cutoff was also determined by calculating the cumulative variance explained by each successive PC. This process continued until the increase in cumulative variance from adding an additional PC was less than 0.001. The PC at which this criterion was met was selected as the maximum number of PCs to include in the model.

The method for identifying the number of significant components in cNMF was applied to all NMF-based methods.^12^ cNMF was executed multiple times, varying the total number of output latent features, denoted as p. The optimal number of latent features, p, was determined by selecting the model that minimized reconstruction error while maximizing the stability or similarity of results across different runs with the same number of latent dimensions.

### Performance accuracy metrics

We employed 2 metrics to quantify the accuracy of inferred cell states against a ground truth vector of cell-state values. To assess the ability of each method to infer a single component that maximally captures cell state, we report the maximum $R^{2}$ between the vector of true cell-state values and the estimated values across all recovered components:

$$\max\;R^{2}=\max\left[R^{2}\left({g,u}_{1}^{\text{ST}}\right),R^{2}\left({g,u}_{2}^{\text{ST}}\right),\ldots ,R^{2}\left({g,u}_{P}^{\text{ST}}\right)\right]$$

where g represents the ground truth cell state activity vector, and $u_{p}^{\mathrm{ST}}$ denotes the p-th component of the state embedding matrixproduced by a given method. The $R^{2}$ is computed as follows:

$$R^{2}\left(g,u\right)=1-\frac{\sum_{i=1}^{I}{\left(g_{i}-u_{i}\right)}^{2}}{\sum_{i=1}^{I}{\left(g_{i}-\bar{g}\right)}^{2}}\text{,}$$

where i indexes each element in the vector g or u with I total elements, respectively.

To evaluate the ability of each method to capture overall cell-state activity, we report the joint adjusted $R^{2}$ from a multivariate regression, where the true cell-state value vector is the dependent variable and the inferred cell states are the independent variables. The $R^{2}$ in this context is computed as:

$$R^{2}=1-\frac{\sum_{i=1}^{n}{\left(g_{i}-\hat{g_{i}}\right)}^{2}}{\sum_{i=1}^{n}{\left(g_{i}-\bar{g}\right)}^{2}}\text{,}$$

where $\hat{g}$ is the prediction outputted by the multivariate regression. The joint adjusted $R^{2}$ is calculated as:

$$\text{Joint}\;\text{Adjusted}\;R^{2}=1-\left(\frac{1-R^{2}}{n-1}\right)\left(\frac{n-1}{n-p-1}\right)\text{,}$$

where n represents the total number of observations and p represents the number of identified states.

In simulations, $R^{2}$ metrics were computed after subsetting to cells putatively expressing the state to distinguish cell-state from cell-type detection. If the state was expressed in a single cell type, then both the embeddings and the ground truth state continuum were subsetted to that specific cell type, and correlations were computed accordingly. Conversely, if the state was expressed across all cell types, the unsubsetted embeddings and state continuum were used.

In the experimental dataset,^31^ we computed $R^{2}$ after subsetting an embedding or a latent feature to a cell type of interest and calculated the corresponding $R^{2}$ for each cell type. Additionally, we used the duration of light exposure as the ground truth cell-state vector for each state. Since the ground truth continuum of the cell state is unknown in the experimental dataset, we assumed a linear relationship between light exposure and latent cell state.

In simulation, metrics for determining the number of components for dimensionality-reduction methods were not used, as the optimal number of PCs or factors was known. In simulations with 7 cell types plus 1 state, 10 PCs were inferred; in simulations with 100 cell types plus 1 state, 105 PCs were inferred. In the experimental dataset, metrics for determining the number of components for dimensionality-reduction methods was used (as described in the above section).

### Cell-type annotation and data preprocessing

The single-cell datasets from Hrvatin et al.^31^ and Morabito et al.^15^ were re-processed using Scanpy.^32^ This involved filtering out cells with high mitochondrial read counts, removing gene outliers to maintain a linear mean-variance relationship, and ensuring that each gene and cell contained a substantial number of reads. Both datasets were originally annotated with cell-type labels as part of their respective studies.

For snATAC-seq datasets, peak data were condensed into a gene activity matrix using the CreateGeneActivityMatrix function in Signac within Seurat.^28^ This function associates peaks with their nearest gene if the peak is located within the gene body or up to 2 kb upstream. The output is a gene-by-cell matrix that can undergo quality control (QC) as previously described, owing to its transformed dimensionality from cells by peaks to cells by genes.

The count-based gene-by-cell matrices were then log-normalized and scaled to ensure a total expression of 10,000 reads per cell (TP10K normalization). Subsequently, the data were standardized to a mean of 0 and a variance of 1 for each gene. Variable genes were selected using the highly_variable_genes function in Scanpy.^32^ Covariates regressed out during the analyses included batch, total sequencing counts per cell, percentage of mitochondrial counts, age, and sex. When ResidPCA was applied, cell type was additionally regressed out.

In the Hrvatin et al.^31^ dataset, we tested both 3,000 and 20,000 variable genes as inputs for each method to assess whether a higher or lower gene threshold resulted in better performance. In the Morabito et al.^15^ analysis, we used the top 20,000 most variable genes to minimize *ad hoc* thresholding and capture all genes in the genome. To determine the number of significant cellular states, we employed the BIC adapted for PCA.^30^

A downsampling analysis of the scRNA-seq expression matrix was conducted using data from Hrvatin et al.^31^ to evaluate the sensitivity of each dimensionality-reduction method in detecting light-induced states with fewer samples. The entire quality-controlled dataset was downsampled to 35,000, 25,000, 15,000, 10,000, and 5,000 cells, respectively, and subsequently used as input for each method. Cells were randomly selected for downsampling, and this process was repeated with 5 different random seed iterations. The BIC method was employed to determine the number of significant states for PCA-based methods, while error-stability optimization was used for NMF-based methods.

In the Morabito et al.^15^ dataset, we quantified the fraction of variation explained by cell type using the following approach. First, we regressed out all covariates except for cell type from the snATAC-seq or snRNA-seq matrix. The resulting cells-by-genes matrix was then standardized. Second, each column of this standardized matrix, representing gene-wise data, was regressed on cell type. We calculated the predicted cell-type gene expression matrix by multiplying the cell type by the recovered beta-coefficients. Finally, the average variance of this predicted matrix was computed and reported as the average variance explained by cell type.

### Heritability enrichment analysis with LDSC-SEG

We investigated AD heritability using established GWAS associations from the latest AD GWAS by Bellenguez et al.^33^ We employed linkage disequilibrium score regression applied to specifically expressed genes (LDSC-SEG),^3^ a methodology designed to partition polygenic trait heritability based on functional annotations so as to quantify the contribution of our identified states to AD heritability. To define a state annotation, we selected the top and bottom 200 genes within each PC-derived state as separate annotations for input into LDSC-SEG. The top and bottom 200 genes were selected because PCA components are mean centered, and directionality is not known. To identify differentially expressed genes associated with specific cell types, we used the Scanpy^32^ rank_genes_groups_df function. The top 200 ranked differentially expressed genes from each cell type were also used as annotations for input into LDSC-SEG. We retained annotations with significant enrichment scores at a false discovery rate (FDR)-corrected *p* value threshold, categorizing states as significantly enriched for AD heritability if they met this criterion.

### Characterizing cell states

To determine whether a given cell type was involved in a cell state, we calculated the maximum squared correlation between each state identified by ResidPCA or standard PCA and the respective cell-type-specific states identified by iterative PCA methods. For example, to assess whether a state derived from ResidPCA was expressed in excitatory neurons, the maximum correlation was calculated between the ResidPCA state and all significant iterative PCA-derived states identified in neurons. The resulting distribution of squared correlations was graphed per cell type to determine a correlation cutoff at the saddle point of the bimodal distribution.

States identified by ResidPCA exceeding this cutoff were considered to be expressed in the corresponding cell type. States were labeled as cell-type specific or agnostic based on the squared correlation between each state and cell-type-specific states identified by iterative PCA methods. Cell-type-agnostic states are those that ResidPCA can detect, whereas state-of-the-art methods such as iterative PCA lack this capability, underscoring the superior ability of ResidPCA to identify a variety of states.

The GSEApy package^34^ in Python was used to compute gene set enrichments. Five collections of gene sets from the Human MSigDB were used: the human hallmark gene sets, human positional gene sets, human curated gene sets, human regulatory target gene sets, and human ontology gene sets. For mouse analyses on the Hrvatin et al.^31^ study, Gene Ontology enrichments from the study were used, along with 2 gene sets from Mouse MSigDB^35^ (mouse-ortholog hallmark gene sets and mouse curated gene sets), and a recently curated dataset containing gene sets related to the mouse nervous system. Additionally, we used a recently published gene set specific to beta-amyloid production.^16^

## Results

### ResidPCA overview

Here, we propose ResidPCA, a method to identify a denoised set of latent cell states from single-cell data, that is efficient and robust to cell-type heterogeneity. The procedure for ResidPCA is as follows: it identifies cell type from single-cell expression data by leveraging established approaches (e.g., marker genes or projection from an atlas). It then regresses out cell-type-driven expression. Finally, it applies PCA to the residualized matrix corresponding to state-driven expression. This method effectively denoises the data by removing cell-type-driven expression, which can obscure accurate cell-state estimation due to variability or noise in gene expression associated with cell identity. As a result, ResidPCA can identify a diverse range of cell states—both rare and common—that are independent of cell type and may be unique to a single cell type or span multiple cell types.

The generative model underlying ResidPCA assumes the log-normalized TP10K gene expression data can be represented as a mixture of 2 multivariate Gaussian distributions with identical variances—one reflecting state-driven expression and the other capturing cell-type-driven expression. Specifically, the model assumes:

$$z_{ij}∼N\;\left(\theta_{ij}^{\mathrm{CT}}+\theta_{ij}^{\mathrm{ST}},\;\sigma_{j}^{2}\right)\text{,}$$

where $z_{ij}$ represents the normalized gene expression for gene j in cell i, $\theta_{ij}^{\mathrm{CT}}$ denotes the cell-type-driven expression component, $\theta_{ij}^{\mathrm{ST}}$ denotes the state-driven expression component, and $\sigma_{j}^{2}$ denotes the variance for gene j.

To estimate the cell-type-driven expression component for gene j in cell i ($\theta_{ij}^{\text{CT}}$), ResidPCA decomposes it as the inner product of the cell-type indicator of cell i, $U_{i,:}^{\mathrm{CT}}$ (the i-th row), and the normalized mean expression vector of gene j across cell types, $V_{j}^{\mathrm{CT}}$ (the j-th column) quantifying how active a gene is in each cell-type-expression profile:

$$\theta_{ij}^{CT}=\langle U_{i,:}^{CT},V_{j}^{CT}\rangle \;.$$

The state-driven component for gene j in cell i ($\theta_{ij}^{\text{ST}}$) is estimated similarly, using the inner product of the cell-state embeddings of cell i, $U_{i,:}^{\mathrm{ST}}$ (the i-th row), which represents the state occupancy of each cell, and the cell-state gene loadings of gene j, $V_{j}^{\mathrm{ST}}$, quantifying how active a gene is in each state:

$$\theta_{ij}^{\mathrm{ST}}=\langle U_{i,:}^{\mathrm{ST}},V_{j}^{\mathrm{ST}}\rangle \text{.}$$

Thus, the normalized expression data $z_{ij}$ can be reparameterized as:

$$z_{ij}∼N\;\left(\langle U_{i,:}^{\mathrm{CT}},V_{j}^{\mathrm{CT}}\rangle +\langle U_{i,:}^{\mathrm{ST}},V_{j}^{\mathrm{ST}}\rangle ,\;\sigma_{j}^{2}\right)\text{.}$$

During inference, ResidPCA first estimates the cell-type-driven expression matrix ${\hat{Z}}^{\mathrm{CT}}\in {\mathbb{R}}^{I\times J}$ (where I is the total number of cells and J is the total number of genes) and then uses ${\hat{Z}}^{\text{CT}}$ to estimate the state-driven expression matrix ${\hat{Z}}^{\mathrm{ST}}\in {\mathbb{R}}^{I\times J}$ from the normalized and standardized TP10K gene expression matrix $Z\in {\mathbb{R}}^{I\times J}$. For each gene j, the cell-type-specific expression component (${\hat{Z}}_{j}^{\text{CT}}$) is estimated via linear regression. This regression models the relationship between the cell-type indicator matrix $U^{\mathrm{CT}}$ and the total normalized and standardized TP10K expression for gene j ($Z_{j}$). The regression coefficients ${\hat{V}}_{j}^{\text{CT}}$ represent the cell-type-driven mean expression of gene j across all cell types:

$$Z_{j}∼U^{\mathrm{CT}}{\hat{V}}_{j}^{\mathrm{CT}}\text{.}$$

Second, to compute the state-driven expression matrix, the estimated cell-type-driven expression component—derived as the reconstruction estimate from the above regression— is subtracted from the total TP10K-normalized expression. This yields an expression component that is uncorrelated with cell type and represents the additive state-driven expression component of gene j across all states:

$${\hat{Z}}_{j}^{\mathrm{ST}}=Z_{j}-U^{\mathrm{CT}}{\hat{V}}_{j}^{\mathrm{CT}}=Z_{j}-{\hat{Z}}_{j}^{\text{CT}},$$

where ${\hat{V}}_{j}^{\mathrm{CT}}$ represents the estimated regression coefficients or betas outputted by the regression for a given gene j.

Third, and finally, the estimated matrix representing the total state-driven expression across all states (${\hat{Z}}^{\mathrm{ST}}$) is decomposed into individual state-specific components. PCA is applied to the covariance matrix of ${\hat{Z}}^{\mathrm{ST}}$ to obtain the state-driven cell embeddings, ${\hat{U}}_{i,:}^{\text{ST}}$, for each cell i, and the state-driven gene loadings, ${\hat{V}}_{j}^{\text{ST}}$, for each gene j, along with the associated eigenvalues ordering states by total variance explained. ResidPCA generates a set of states that captures both how strongly each cell expresses each state (${\hat{U}}^{\text{ST}}$) and which genes regulate each state (${\hat{V}}^{\text{ST}}$).

### Benchmarking ResidPCA in simulation

We compare ResidPCA to multiple well-established dimensionality-reduction methods: standard PCA, which identifies primary and global sources of variation in data by projecting it onto new, orthogonal axes representing the most significant directions of variance^36^; NMF, which produces non-orthogonal components that are non-negative and represent additive combinations of the original single cell count data^37^; cNMF, an extension of NMF, which improves the stability of NMF by consolidating the latent representations across multiple runs of NMF^12^; and scaled NMF, which regresses out covariates from a positive count matrix and enforces non-negativity by setting any negative values to zero before inputting the matrix into the NMF algorithm^38^^,^^39^ (Tables 1 and 2). Finally, UMAP is also used, which is the traditional method for visualizing single-cell data that aims to preserve both global and local structure in the data.^40^ Although recent findings indicate that UMAP does not consistently preserve these high-dimensional structures in its 2-dimensional representation, we include it as a measure of what users might expect to see from typical visual inspection of the data.^41^

**Table 1:** Overview of various benchmarked methods used for detecting cell states

Method	Description
ResidPCA	an extension of PCA tailored for identifying cell states in single-cell data, where cell-type effects are regressed out from the data matrix, allowing the recovered orthogonal components to represent a denoised set of cell states not confounded by cell-type-driven noise in expression
Standard PCA	a dimensionality-reduction method that transforms a matrix into a set of orthogonal components, capturing the maximum variance in the data, with fewer dimensions
Iterative PCA	PCA applied iteratively to a subset of the single-cell data specific to each cell type, effectively identifying a set of cell-type-specific states for each individual cell type
NMF	a factorization method that decomposes a matrix into non-negative components
cNMF	an extension of NMF that incorporates multiple factorizations of NMF to identify stable and robust components in data
Scaled NMF	an extension of NMF that enables the regression or removal of covariates by first regressing out the covariate effects from single-cell data and then setting any resulting negative values to zero to maintain non-negativity
UMAP	a dimensionality-reduction method that preserves the local structure of the data while reducing its dimensionality for visualization purposes

cNMF, consensus non-negative matrix factorization; NMF, non-negative matrix factorization; PCA, principal-component analysis; UMAP, uniform manifold approximation and projection.

**Table 2:** Comparison of various benchmarked methods used for detecting cell states

Feature	ResidPCA	Standard PCA	Iterative PCA	NMF	cNMF	Scaled NMF	UMAP
Linear vs. nonlinear	linear	linear	linear	linear	linear	linear	non-linear
Orthogonality constraint	yes	yes	yes	no	no	no	no
Non-negativity constraint	no	no	no	yes	yes	yes	no
Preservation of global structure	high	high	high	moderate	moderate	moderate	low
Scalability to large datasets	high	high	high	moderate	moderate	moderate	high
Applied to entire dataset	yes	yes	no (applied iteratively to each cell type)	yes	yes	yes	yes
Findings from our work	high sensitivity across a broad range of state types, including those spanning multiple cell types and those restricted to a single cell type; robust even with many cell types present and states are rare; useful for cell type discovery or when cell types known to express the cell state are unknown	performs well for common, globally expressed states, but sensitivity declines for rare states and when many cell types are present	improved detection for cell-type-specific states, but limited for states spanning multiple cell types	performs well for common states when the set of cell types expressing the state is known, but sensitivity declines for rare states and when many cell types are present	similar to NMF, relies on a heuristic for determining the number of states to retain and shows high variability with small changes to the number of states output	poor performance overall, likely due to loss of useful information when zeroing out negative values	captures cell types well but does not robustly capture cell states

**Figure 1:**
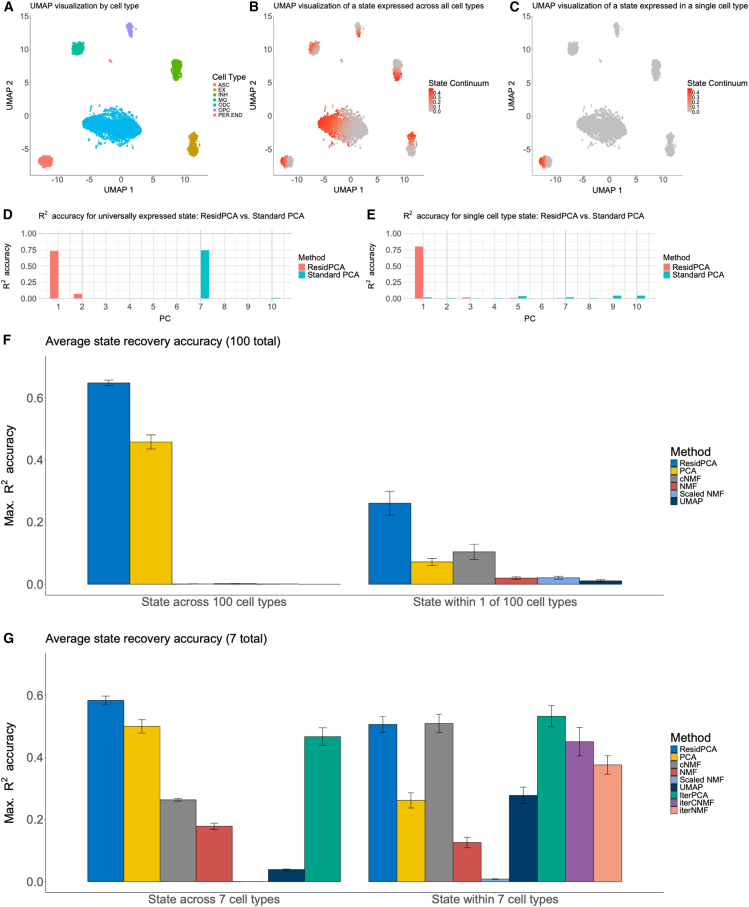
Benchmarking ResidPCA against well-established methods in simulation. (A–C) UMAP visualizations of representative simulations, colored by (A) cell type, (B) a state expressed across all 7 cell types, and (C) a state restricted to a single cell type. (D and E) Bar plots showing the *R*^*2*^ accuracy in 1 representative simulation between the ground truth simulated state continuum and the corresponding PC embeddings identified by ResidPCA or standard PCA. Results are shown for (D) a state expressed across all 7 cell types and (E) a state expressed in a single cell type. To ensure fair comparisons, *R*^*2*^ correlations were computed only after subsetting both the ground-truth state and the recovered embedding to the cell type(s) capable of expressing the state, thereby avoiding inflated accuracy from correlations between cell identity and state. (F and G) Bar plots of average simulation accuracy for each method in recovering the true simulated state continuum, aggregated across parameter settings. Simulations varied 2 parameters, the fraction of genes and the fraction of cells expressing the state, and for each parameter combination, *R*^*2*^ metrics were averaged across 10 seeds until the standard error fell below 0.085. Each parameter configuration was replicated 10 times. Maximum average *R*^*2*^ accuracy is shown for states expressed across all cell types (left) and within a single cell type (right) in simulations with (F) 100 or (G) 7 total cell types. Error bars indicate one standard error of the mean.

We systematically evaluated the accuracy of ResidPCA compared to well-established methods in recovering cell states from simulated data. Single-cell datasets were generated using realistic parameters learned from real data (material and methods; Figures 1A and S1) and simulated to reflect 2 scenarios: a state expressed in a subset of the total cell population across all cell types (Figure 1B) and a state exclusively expressed in a subset of a single cell type (Figure 1C). Simulations used either 7 or 100 cell types to test performance across varying heterogeneity, under the assumption that cell type and state are independent. In Figure 1B, we illustrate an example of a simulated state that is expressed across all 7 cell types. Notably, ResidPCA demonstrates superior sensitivity by detecting this state in its first PC, while standard PCA identifies the state in a later PC (PC7) (Figure 1D). Likewise, in Figure 1C, we present an example of a simulated state that spans only 1 of the 7 cell types. Here, we again see ResidPCA has more sensitivity by identifying the state in the second PC, whereas standard PCA fails to capture it in any single PC (Figure 1E).

For each method, we then quantified the accuracy of the state detection in each simulation by computing the maximum *R*^*2*^ between each inferred component and the true state continuum. The maximum was used (as opposed to the *R*^*2*^ with the “first” component) because some methods produce an arbitrary ordering of inferred components. For each simulated state, only cell types in which the state was putatively active were considered when quantifying accuracy, to distinguish cell-state from cell-type detection, meaning that if the state was expressed in a single cell type, then both the embeddings and the ground truth state continuum were subsetted to that specific cell type, and correlations were computed accordingly. Conversely, if the state was expressed across all cell types, the unsubsetted embeddings and state continuum were used to compute the correlations.

ResidPCA consistently demonstrated higher accuracy compared with other methods in simulations, particularly when cell-type heterogeneity was high or states spanned multiple cell types. We simulated an intentionally high cell-type heterogeneity scenario consisting of 100 total cell types; ResidPCA had higher accuracy than all other methods, regardless of whether states were expressed within a single cell type or across all cell types (Figure 1F). For simulations with states active across all cell types, ResidPCA achieved an average *R*^*2*^ of 0.65 (95% CI: 0.62–0.67), compared to an average *R*^*2*^ of 0.46 (CI: 0.43–0.49) for standard PCA and *R*^*2*^ < 0.0021 (upper 95% CI < 0.0025) for all other methods (cNMF, NMF, and UMAP) (Figure 1F). For simulations with states active in 1/100 total cell types, all methods had reduced performance compared with ResidPCA, with a mean *R*^*2*^ of 0.26 (95% CI: 0.22–0.30) compared with a mean *R*^*2*^ of 0.11 (95% CI: 0.10–0.13) for cNMF and 0.088 (95% CI: 0.075–0.10) for standard PCA (Figure 1F). Across all methods, overall performance improved with increased state abundance (defined as states with a high fraction of cells and genes expressing the state), exemplified by the average difference in accuracy between an abundant versus a rare cell state (ResidPCA Δ*R*^*2*^ = 0.63, standard PCA Δ*R*^*2*^ = 0.70, cNMF Δ*R*^*2*^ = 7.8 × 10^−4^, NMF Δ*R*^*2*^ = 6.4 × 10^−3^, scenario: 1 state expressed across 100 cell types) (Figure S1). ResidPCA was consistently more sensitive compared to alternative methods, requiring fewer cells and genes expressing the simulated state to detect states effectively; for instance, in simulations where the state was present within 1/100 cell types, and with a fixed total number of genes expressing the state, standard PCA (the next best-performing method) required over 4.7 times more cells to attain an accuracy similar to that of ResidPCA (Figure S1).

In simulations with lower cell-type heterogeneity or a smaller number of cell types (7), ResidPCA was still the most accurate method, although differences in performance between ResidPCA and standard PCA were less pronounced. In simulations with a state spanning all 7 cell types, ResidPCA performed better on average than standard PCA (mean *R*^*2*^ = 0.58 [95% CI: 0.56–0.61] and 0.50 [95% CI: 0.46–0.54], respectively), and both methods substantially outperformed cNMF (mean *R*^*2*^ 0.26 [95% CI: 0.26–0.27]) (Figure 1G). ResidPCA outperformed standard PCA when the percentage of genes in the state was low or the state was rarer but was otherwise comparable (Figure S1). In simulations with a state spanning 1/7 cell types, ResidPCA (mean *R^2^* = 0.51 [95% CI: 0.46–0.56]) had performance comparable to that of cNMF (mean *R*^*2*^ = 0.51 [95% CI: 0.45–0.57]) and substantially outperformed standard PCA (mean *R*^*2*^ = 0.26 [95% CI: 0.21–0.31]) (Figure 1G). Overall performance again improved with increased state abundance in states across 7 cell types, exemplified by the average difference in accuracy between an abundant versus rare cell state per method (ResidPCA Δ*R*^*2*^ = 0.83, standard PCA Δ*R*^*2*^ = 0.76, cNMF Δ*R*^*2*^ = −0.084, NMF Δ*R*^*2*^ = 0.24, scenario: 1 state expressed across 7 cell types) (Figure S1).

We next evaluated the ability of different methods to capture cell-state signals across all significant recovered components by examining the joint adjusted *R^2^*, which quantifies overall detectability of the cell-state signal with the recovered component space. In contrast, our previous analysis focused on the maximum *R^2^*, which measures how well a method consolidates the cell-state signal into a single component rather than dispersing it across multiple components. We found that cNMF slightly outperformed ResidPCA and standard PCA in a state spanning 7 cell types (cNMF joint adjusted *R*^*2*^ = 0.68 [95% CI: 0.66–0.71], ResidPCA joint adjusted *R*^*2*^ = 0.64 [95% CI: 0.61–0.67], PCA joint adjusted *R*^*2*^ = 0.52 [95% CI: 0.48–0.56]) (Figure S1). However, in capturing the true state with a single component (shown previously), NMF-based methods exhibited decreased accuracy compared to PCA-based methods (ResidPCA maximum *R*^*2*^ = 0.58, PCA maximum *R*^*2*^ = 0.50, cNMF maximum *R*^*2*^ = 0.26) (Figure 1G). This suggests that NMF-based methods are more prone than PCA-based methods to splitting single states into multiple components. Additionally, the 2 UMAP dimensions generally do not capture a linear correlation with cell state, except in straightforward cases where the state is abundant, cell-type heterogeneity is low, and the state is confined to a single cell type (Figure S1).

Because iterative approaches—where analyses are repeated within 1 cell type at a time—are commonly used in single-cell analysis, we next compared ResidPCA to iterative PCA (IterPCA), iterative NMF (IterNMF), and iterative cNMF (IterCNMF) in our simulation-based benchmarking using the maximum *R*^2^ metric. ResidPCA outperformed iterative methods in detecting a shared state spanning all seven simulated cell types (mean *R*^*2*^: ResidPCA = 0.8 [95% CI: 0.56–0.61], standard PCA = 0.50 [95% CI: 0.46–0.54], and iterative PCA = 0.47 [95% CI: 0.41–0.52]) and performed comparably to iterative PCA for states confined to a single cell type (mean *R*^*2*^: ResidPCA = 0.51 [95% CI: 0.46–0.56] and iterative PCA = 0.53 [95% CI: 0.47–0.60]) (Figure 1G). Iterative methods involve performing PCA, NMF, or cNMF, respectively, on matrices subsetted to the cell type putatively capable of expressing the given state. Unlike methods that require combining iterative PCA to identify within-cell-type states with a separate approach for capturing states spanning multiple cell types—followed by *post hoc* reconciliation of overlapping programs—ResidPCA provides a unified, interpretable solution that captures both within- and across-cell-type variation without any loss in accuracy.

All simulations described thus far assume that cell type and cell state are independent. To assess ResidPCA’s performance when this assumption does not hold, we next evaluated scenarios in which cell type and state are correlated (Figure S2; Note S1). Across a broad range of correlation strengths, ResidPCA consistently matched or outperformed standard PCA, with performance only declining when correlations approached values indistinguishable from pure cell-type differences.

Overall, ResidPCA most accurately recovered simulated states in the majority of instances compared to other methods, particularly in single-cell datasets with a high number of cell types or when states spanned all cell types. ResidPCA was rarely less accurate than standard PCA, suggesting it can serve as a reliable substitute when cell types are known. There were a small number of scenarios when ResidPCA was less accurate than cNMF, primarily when the number of cells and the number of genes in a state was small and all methods had low accuracy.

### Benchmarking ResidPCA against state-of-the-art methods in data with experimentally induced states

To assess the effectiveness of methods in real data, we turned to an scRNA-seq dataset with an experimentally induced state. After re-processing the dataset from Hrvatin et al.^31^ with additional QC, 43,408 cells were retained. In this study (previously used to benchmark cNMF^12^), mice were exposed to varying durations of light, which has been shown to lead to a transcriptional state change in the mouse visual cortex.^31^ We used the amount of light exposure (hours) as a proxy for the true cell state when evaluating the performance of the 7 state detection methods also evaluated in simulations (ResidPCA, standard PCA, IterPCA, cNMF, NMF, scaled NMF, and UMAP). Light exposure may also influence the relative cell-type abundances; therefore, to quantify the ability of the inferred components to capture light-induced states rather than cell-type variation, we calculated *R*^*2*^ accuracy with light exposure within each classified cell type. We additionally evaluated the impact of using a small subset (3,000) of variable genes versus nearly all (20,000) genes on performance, which could not be tested in simulations without imposing specific biological assumptions.

**Figure 2:**
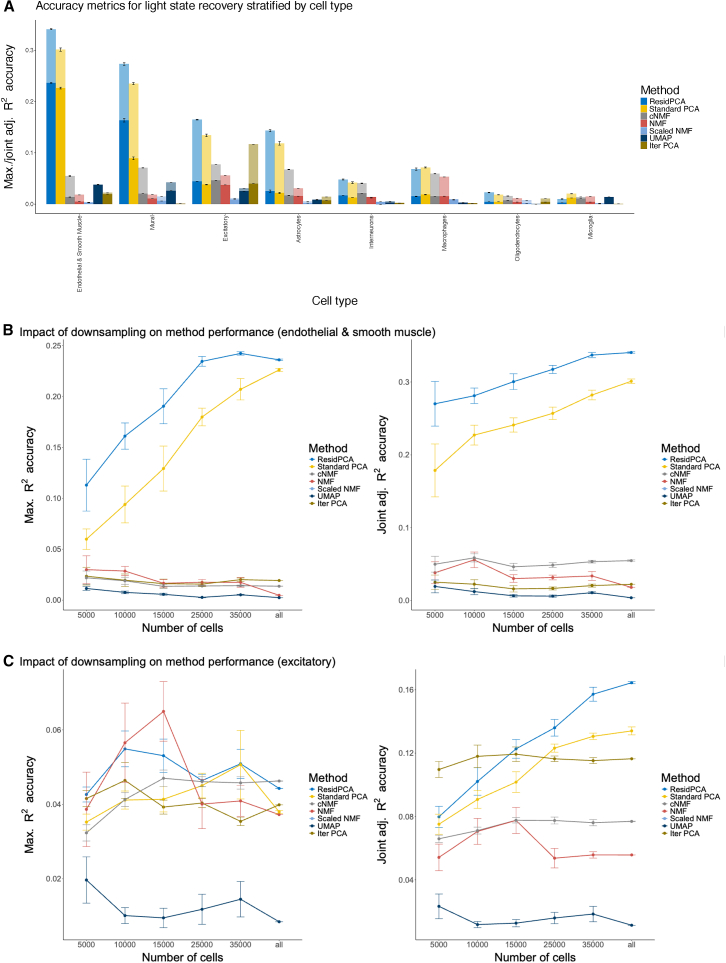
Benchmarking ResidPCA against well-established methods in data with an experimentally induced state (A) Bar plots show 2 accuracy metrics across each cell type: (1) the maximum *R*^*2*^ between any significant state recovered by a given method and light exposure duration (solid bars) and (2) the joint adjusted *R*^*2*^ between all significant states recovered by that method and light exposure duration (transparent bars). (B and C) Accuracy curves are shown for each metric (left: maximum *R*^*2*^; right: joint adjusted *R*^*2*^ under incremental and random downsampling of total cells (*x* axis). Downsampling results are displayed for endothelial and smooth muscle (B) and excitatory neurons (C). Each downsampling condition was repeated 5 times; curves represent averages across replicates, with standard errors shown. All analyses used 3,000 variable genes. We note that for PCA-based methods, the number of significant states was determined by the Bayesian information criterion (BIC) cutoff,^30^ while for NMF-based methods, significance was defined by stability/error maximization^12^ (see material and methods). Error bars indicate one standard error of the mean.

ResidPCA consistently outperformed other methods in recovering state changes across cell types, particularly vascular cell types (endothelial and smooth muscle cells [grouped cell types] and mural cells), which were found to be among the most transcriptionally affected by light exposure, second only to excitatory neurons in the original publication.^31^ Using 3,000 variable genes, ResidPCA achieved a maximum *R*^*2*^ of 0.24 (95% CI: 0.23–0.24) for endothelial and smooth muscle cells, and a maximum *R*^*2*^ of 0.16 (95% CI: 0.16–0.17) for mural cells; cNMF and NMF showed negligible detection in both cell types (Figure 2A). The performance of ResidPCA and cNMF were more similar for excitatory neurons, with maximum *R*^*2*^s of 0.044 and 0.046, respectively (with negligible CIs) (Figure 2A). However, ResidPCA achieved a substantially higher joint adjusted *R*^*2*^ of 0.16 (95% CI: 0.16-0.17) compared to 0.077 (negligible CI) for cNMF (Figure 2A). This supports the hypothesis that excitatory neurons, the cell type most affected by light, induce multiple state changes in response to light exposure that can be captured across multiple distinct components. Our results differ from prior analyses of these data, which only assessed performance after subsetting the input data to excitatory neurons and interneurons (whereas we use the full dataset, including all cell types as input).^12^ Consequently, cNMF may be less effective for the initial exploration of cell states when the target cell type(s) expressing a state are unknown, whereas ResidPCA excels at detecting such states. In all analyses, ResidPCA outperformed standard PCA and cNMF outperformed NMF (Figure 2A). Our findings indicate that ResidPCA effectively identifies unique light-induced states activated in each cell type and can properly recover distinct cell-type-specific states into separate PCs (Notes S2 and S3). This observation aligns with the conclusions of Hrvatin et al.^31^ that each cell type activates a distinct cell state or set of genes in response to light exposure.

Next, we downsampled the number of cells to determine how each method performed under more data-limited or underpowered scenarios. ResidPCA was the leading method in 54% of downsampled analyses in terms of maximum *R*^*2*^ and in 81% of downsampled analyses in terms of joint adjusted *R*^*2*^. ResidPCA was substantially more sensitive for endothelial and smooth muscle cell types, where standard PCA required 2.5 times as many cells to detect light-induced states at the same maximum *R*^*2*^ accuracy, while NMF-based methods never achieved as high a level of accuracy as PCA-based methods (Figure 2B; Table 3, first row). Likewise, for excitatory neurons, standard PCA required 1.7 times as many cells, while cNMF never achieved the same *R*^*2*^ accuracy as ResidPCA (Table 3, second and third rows) (Figure 2C). While NMF may appear largely robust to downsampling, as shown in Figure 2B, this apparent stability is likely misleading. The method performs poorly overall, and the weak correlation with light exposure is likely driven by background noise rather than meaningful signal, resulting in consistently low performance regardless of downsampling. Finally, iterative PCA consistently underperformed in identifying light-induced, cell-type-specific states. It never matched the accuracy of ResidPCA, even with an increased number of cells or samples (Table 3, fourth and fifth rows) (Figures 2B and 2C). This underperformance of iterative PCA likely arises from the fact that using only a subset of the data reduces sensitivity for states that span multiple cell types (even if they are dominant in 1 cell type). We also investigated the sensitivity of each method to the number of genes included in the analysis. ResidPCA exhibited the smallest variability in accuracy relative to the number of variable genes included in the analysis, but no single optimal gene count emerged as universally the best (Figure S3; Note S4).

**Table 3:** Results from downsampling analysis comparing the number of cells required to achieve specific accuracy thresholds for each method

3,000 var genes achieved *R*^*2*^ accuracy of	Cell type	ResidPCA	Standard PCA	cNMF	NMF	Scaled NMF	Iterative PCA
Maximum *R*^*2*^ ≥ 0.16	endothelial and smooth muscle	10,000 cells; *R*^*2*^ = 0.16 (0.14–0.19)	25,000 cells; *R*^*2*^ = 0.18 (0.16–0.20)	∗*R*^*2*^ < 0.022 in all scenarios	∗*R*^*2*^ < 0.030 in all scenarios	∗*R*^*2*^ < 0.011 in all scenarios	∗*R*^*2*^ < 0.024 in all scenarios
Maximum *R*^*2*^ > 0.050	excitatory neurons	10,000 cells; *R*^*2*^ = 0.055 (0.045–0.064)	35,000 cells; *R*^*2*^ = 0.051 (0.033–0.067)	∗*R*^*2*^ < 0.047 in all scenarios	10,000 cells; *R*^*2*^ = 0.057 (0.036–0.077)	∗*R*^*2*^ < 0.020 in all scenarios	∗*R*^*2*^ < 0.042 in all scenarios
Joint adjusted *R*^*2*^ ≥ 0.12	excitatory neurons	15,000 cells; *R*^*2*^ = 0.12 (0.11–0.13)	25,000 cells; *R*^*2*^ = 0.12 (0.12–0.13)	∗*R*^*2*^ < 0.078 in all scenarios	∗*R*^*2*^ < 0.078 in all scenarios	∗*R*^*2*^ < 0.023 in all scenarios	∗*R*^*2*^ < 0.023 in all scenarios
Maximum joint adjusted *R*^*2*^	endothelial and smooth muscle	all cells; *R*^*2*^ = 0.34 (0.34–0.34)	all cells; *R*^*2*^ = 0.30 (0.30–0.31)	∗*R*^2^ < 0.058 in all scenarios	∗*R*^*2*^ < 0.056 in all scenarios	∗*R*^*2*^ < 0.019 in all scenarios	∗*R*^*2*^ < 0.025 in all scenarios
Maximum joint adjusted *R*^*2*^	excitatory neurons	all cells; *R*^*2*^ = 0.16 (0.16–0.17)	all cells; *R*^*2*^ = 0.13 (0.13–0.14)	∗*R*^*2*^ < 0.078 in all scenarios	∗*R*^*2*^ < 0.078 in all scenarios	∗*R*^*2*^ < 0.023 in all scenarios	∗*R*^*2*^ < 0.023 in all scenarios

The first 2 rows report the minimum number of cells needed to reach a maximum *R*^*2*^≥0.16 in endothelial and smooth muscle cells and *R*^*2*^≥0.050 in excitatory neurons, respectively. The third row indicates the cell count required to achieve a joint adjusted *R*^*2*^≥0.12 in excitatory neurons. The final 2 rows summarize the maximum joint adjusted *R*^*2*^ attained by each method in endothelial and smooth muscle cells. An asterisk (∗) marks methods that did not reach the specified accuracy threshold. Confidence intervals correspond to the 95% confidence level.

To further evaluate ResidPCA on data with a different pseudo-ground truth, we applied it to an snATAC-seq dataset from Morabito et al.,^15^ which includes both high-level and granular brain cell-type annotations. We note that these subtypes were identified using a combination of marker genes, manual inspection, and hierarchical clustering and are therefore expected to be partially but not entirely correlated with broad cell types and may also be biased toward visual coherence. ResidPCA was run using broad cell-type labels (e.g., excitatory neurons, inhibitory neurons, oligodendrocytes) as covariates, effectively regressing out variation attributable to these major classes. We then evaluated whether the resulting components captured fine-scale cell types/states by computing correlations between the ResidPCA components and granular subtype labels within each broad class. For comparison, standard PCA without adjustment for cell type and iterative PCA (PCA performed on cell-type-specific data subsets) were applied. In short, we find that ResidPCA often identifies these states with slightly higher sensitivity than standard PCA and with much higher sensitivity than iterative PCA (Figure S4A).

We first identified, for each granular cell state, the PC from ResidPCA, standard PCA, or iterative PCA that yielded the highest significant correlation with each granular cell type separately. Across all 33 granular states, ResidPCA produced the highest significant correlation in 15 clusters, standard PCA in 14, and iterative PCA in 4 (Figure S4B). This strongly validates ResidPCA, showing that analyzing all cells together can provide greater power than iterative PCA, even for cell-type-specific analyses. ResidPCA produced the strongest correlations in the majority of astrocyte and excitatory neuron granular state clusters—specifically, in 8 out of 11 such clusters (Figure S4A). Notably, the states where standard PCA performs better tend to be those with relatively low correlations to any PC—such as certain sub-states in oligodendrocytes—suggesting that standard PCA may offer slight advantages in weakly identifiable states (or that these manually defined subtypes may have high uncertainty). In contrast, for states with stronger correlations, ResidPCA generally outperforms the other methods.

These results demonstrate that ResidPCA can recover biologically meaningful intra-type structure associated with finer subtypes, even when the dominant variation attributable to broad cell-type distinctions, often correlated with these subtypes, is explicitly regressed out. This supports the use of ResidPCA in contexts where fine-grained heterogeneity is of interest but only coarse annotations are available or desirable for modeling purposes.

### ResidPCA uncovers diverse state types: From broad cell-type associations to unique or unassigned states

**Figure 3:**
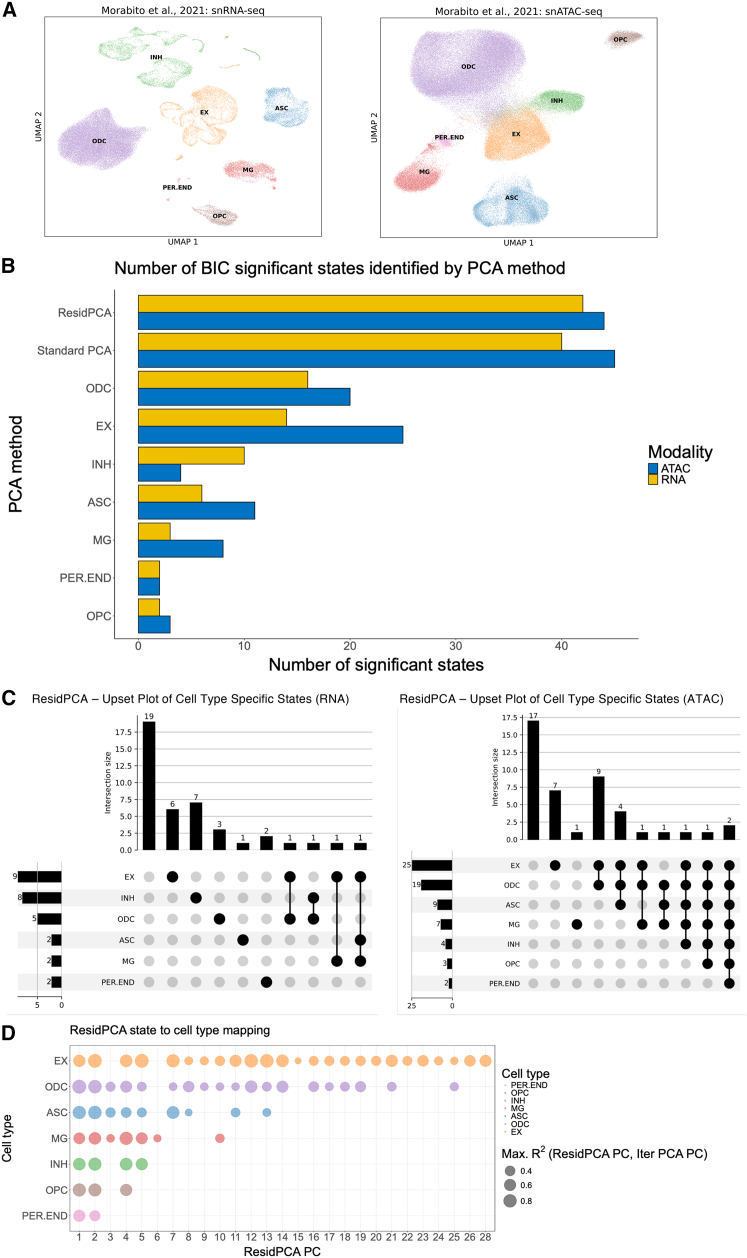
ResidPCA uncovers cell-type-specific and cross-cell-type states underlying Alzheimer's disease heterogeneity (A) UMAP visualizations of re-QC data from Morabito et al.,^15^ showing gene expression from snRNA-seq (left) and peak accessibility from snATAC-seq (right). ResidPCA and 2 established methods—standard PCA and iterative PCA—were applied independently to each modality. (B) Bar plot summarizing the number of BIC-significant states identified per modality for each PCA method. The *y* axis labels with cell-type abbreviations denote iterative PCA performed on data restricted to that specific cell type. (C) Upset plots showing the frequency and distribution of ResidPCA-recovered states across cell types in RNA (left) and ATAC (right) data. States are categorized by whether they are expressed across all, multiple, single, or no cell types. (D) Bubble plot showing the maximum *R*^*2*^ correlation between each ResidPCA state (*x* axis) and all BIC-significant iterative PCA states within each cell type. Only *R*^*2*^ values >0.20 are displayed. Bubble size reflects correlation magnitude. The plot highlights the correspondence of ResidPCA-derived states to cell-type-specific states detected by iterative PCA (see material and methods). ASC, astrocytes; EX, excitatory neurons; INH, inhibitory neurons; MG, microglia; ODC, oligodendrocytes; OPC, oligodendrocyte precursor cells; PER.END, pericytes/endothelial cells.

To demonstrate their utility in characterizing biological variation, we applied PCA-based methods, including ResidPCA, to a large multi-modal single-nuclei dataset from late-stage AD patients, which we re-processed with additional QC, resulting in datasets consisting of 61,471 and 130,377 cells for RNA-seq and ATAC-seq, respectively (Figure 3A, see material and methods).^15^ We chose this dataset due to several key factors. First, single-cell studies have increasingly focused on brain-derived data because of the brain’s vast cellular heterogeneity and intricate spatial organization.^9^^,^^15^ Recent advances have identified over 3,000 distinct cell types and states in the human brain, underscoring its complexity.^9^^,^^20^^,^^42^^,^^43^ Second, the growing prevalence of neuroinflammatory disorders, particularly AD, coupled with the known polygenicity and high heritability of complex brain disorders like AD, as highlighted by large GWAS studies, makes this dataset particularly relevant for understanding the molecular and cellular mechanisms underlying AD.^33^

When analyzing sc-/snATAC-seq data, which are typically very sparse, peak data were mapped to the nearest gene of interest, since this has previously been shown to improve the signal-to-noise ratio in this modality^44^ while enabling consistent state comparisons with sc-/snRNA-seq. ResidPCA conditioned out the effect of cell type on gene expression, removing approximately 15% and 20% of the variation in snATAC and snRNA expression, respectively, in the Morabito et al. datasets^15^ (see material and methods). The BIC adapted for PCA (see material and methods), was applied to ResidPCA-identified states, revealing 42 and 44 significant cell states in RNA-seq and ATAC-seq data, respectively (Figure 3B). The BIC-significant states identified by ResidPCA explained 15% of the cumulative variance in the residualized matrix for the RNA dataset and 6% for the ATAC dataset (Figure S5).The BIC method yielded many more significant states for both RNA and ATAC than the elbow plot method (RNA: 4 states, ATAC: 5 states), suggesting much higher dimensionality in the data than is typically considered (Figure S5). Among the ResidPCA-identified ATAC-based states, 2 states spanned all cell types (states correlated with all cell types), 17 states exhibited specificity by spanning multiple but not all cell types, 8 states were expressed in only a single cell type, and 17 states were cell-type agnostic or could not be mapped to a specific cell type. Note that cell type agnostic states identified by ResidPCA are not detectable using cell-type-specific methods such as iterative PCA (Figure 3C) (see material and methods). Standard PCA may identify more significant states than ResidPCA because it captures both cell-type and cell-state variation (e.g., 5 cell types plus 5 states may yield 10 PCs, whereas ResidPCA isolates only the 5 states). The overlap between states identified by the 2 methods is only moderate (average maximum squared correlation = 0.55), with much of the similarity arising from sparsity-driven shared low-activity regions rather than a consistent biological signal (Figure S6). More broadly, we also observed a general lack of consistency between states identified from RNA- and ATAC-based data, consistent with recent literature (Figure S6).

Since ATAC data were the modality that generated cell states expressed in all known cell types, we functionally interpreted those 2 states, which corresponded to the top PCs identified by ResidPCA, explaining the greatest variance in ATAC expression (Figures 3C and 3D). Using gene set enrichment analysis (GSEA), we found that PC1 was associated with an interconnected set of pathways that contribute to disease pathology through various mechanisms including GABA receptor complex (FDR *p* value: *p* < 1.2e−5, normalized enrichment score [NES]: 3.03), metabolic pathways like steroid hormone biosynthesis (FDR *p* value: *p* < 1.2e−5, NES: 2.6) and glucuronidation (FDR *p* value: *p* < 1.2e−5, NES: 2.54), and immune response pathways including type I interferon receptor binding (FDR *p* value: *p* < 1.2e−5, NES: 2.7). Dysregulation of GABA receptor complexes can lead to imbalanced neurotransmission and increased excitotoxicity, which may exacerbate neurodegenerative processes in AD,^45^^,^^46^ while alterations in steroid hormone biosynthesis impact brain function and neuroprotection, potentially interacting with neurotransmitter systems and inflammatory responses.^47^ Both neurons and astrocytes produce cholesterol, a hormone strongly implicated in AD, supporting the idea that the state identified by ResidPCA involves multiple cell types.^48^ Impaired glucuronidation affects the detoxification and clearance of neurotoxic substances such as amyloid-beta, which can exacerbate neuroinflammation and influence hormonal and neurotransmitter pathways,^49^^,^^50^ while chronic inflammation mediated by type I interferons promotes neuroinflammation mediated by microglia and endothelial cells, which might disrupt both hormonal balance and neurotransmitter systems, creating a feedback loop that accelerates AD progression.^51^^,^^52^ Thus, these pathways collectively contribute to AD through a variety of cell types by affecting neurotransmission, neuroinflammation, and the metabolism of neurotoxic substances.

In contrast, PC2 predominantly reflects immune response pathways, including peptide antigen assembly with major histocompatibility complex (MHC) protein complex (FDR *p* value: *p* < 1.2e−5, NES: 1.9) and antigen processing and presentation of peptide antigen via MHC class I (FDR *p* value: *p* < 1.2e−5, NES: 1.9). These pathways play key roles in the immune response and neural plasticity, while key genes within the MHC class I protein complex have been implicated in the risk of AD.^53^^,^^54^ Peptide antigen assembly, processing, and presentation via MHC class I involves the formation of peptide-MHC complexes, which are crucial for the immune system to recognize and eliminate cells presenting endogenous antigens, including protein aggregates.^55^ In AD, these pathways are particularly relevant because dysregulation of immune responses and altered antigen presentation contribute to neuroinflammation and disease progression, especially implicated in microglia.^56^ However, given the systemic nature of inflammation and the fundamental role of MHC class I in cellular antigen presentation, these pathways are likely detectable across all cell types, as immune signaling and antigen processing are core processes shared broadly within the cellular environment. Specifically, the aggregation of amyloid-beta impairs antigen presentation by destabilizing the neuronal MHC class I complex.^57^ This leads to inadequate immune surveillance and the accumulation of toxic proteins,^57^ such as amyloid-beta, as well as dendritic atrophy,^58^ which are hallmarks of AD pathology. Consequently, disruptions in these pathways exacerbate neuroinflammation and impair neuronal function, thereby influencing the development and progression of AD.

### Identifying key states enriched for AD heritability enrichment

**Figure 4:**
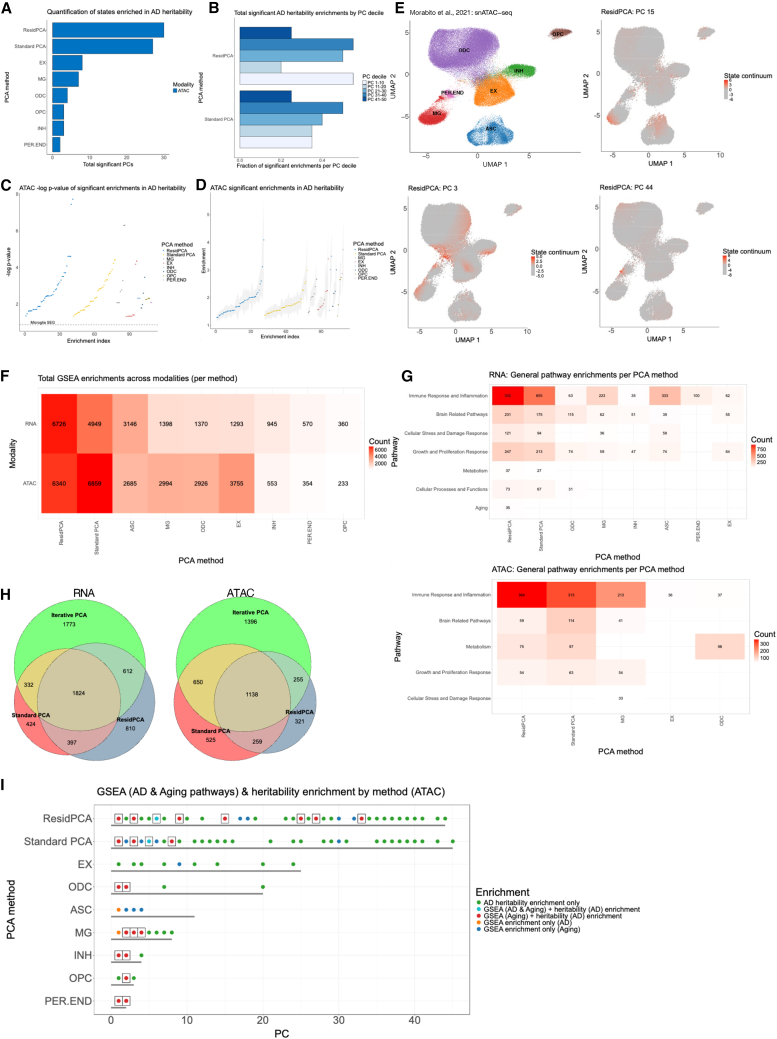
Mapping Alzheimer's disease heritability enrichment across PCA-derived cellular states. (A) Number of BIC-significant states identified by ResidPCA, standard PCA, and iterative PCA that are enriched for AD heritability (FDR <0.05) in both RNA and ATAC data from Morabito et al.^15^ (B) Fraction of significant ATAC-derived states enriched for AD heritability across PC deciles (10 PCs per decile) in standard PCA and ResidPCA. (A) and (B) show discrete counts of significant enrichments; error bars are not applicable. (C and D) (C) –log10 *p* values and (D) heritability enrichment estimates for all significant ATAC-derived states enriched for AD heritability across PCA methods. The corresponding value for the microglial annotation—the most enriched cell type for AD heritability—is shown as a dashed reference line in (C). (E) UMAP visualization of a subset of ATAC-derived states identified by ResidPCA. (Top left) Colored by cell type, while the remaining images highlight state occupancy, with colors reflecting the embedding loadings for individual states. (F) Heatmap showing the number of significant gene set enrichments per method in both RNA- and ATAC-derived states (FDR <0.005). (G) Heatmaps of significantly enriched pathways grouped into broader biological categories, per method and modality. Only categories with >25 enrichments in at least 1 method are shown. (H) Euler plots showing overlaps in gene set enrichments across PCA methods in RNA (left) and ATAC (right) data (FDR <0.005). For iterative PCA, enrichments represent the union across all 7 cell-type-specific runs. (I) Colocalization analysis of ATAC-derived BIC-significant states enriched for AD heritability, AD-related gene sets, and/or aging-related gene sets. Boxed colocalizations represent states enriched for AD heritability and at least 1 gene set. Gray bars indicate the total number of significant states per method. We note that (H) reflects unique pathways, whereas (F) and (G) report total pathway counts, including non-unique pathways.

We next aimed to connect PCA-based states to the genetic etiology of AD through integration with AD GWAS data. We used LDSC-SEG^3^ to detect disease-related states that contained genes enriched for AD heritability (see material and methods). Overall, only ATAC-based states showed significant heritability enrichment, while RNA-based states did not (Figure 4A), consistent with recent work reflecting a lack of consistency between the ATAC and RNA modalities (Figure S7).^3^^,^^59^^,^^60^^,^^61^ Within the ATAC modality, ResidPCA recovered the most states enriched for AD heritability (ResidPCA: 30 states, standard PCA: 27 states, iterative PCA applications: <9 states) (Figure 4A), with significant enrichments observed in both early and late PCs (i.e., not just those that explained the most variance) (Figure 4B). As a benchmark, we compared heritability enrichments to the AD heritability enrichment for genes specific to microglial cells, the established cell type most enriched for AD heritability.^62^ Strikingly, ResidPCA captured states with the most significant *p* values (minimum *p* value <1.9 × 10^−8^, FDR corrected) and largest heritability enrichments (maximum enrichment = 4.1), with all PCA methods identifying some states that were more enriched than the microglia annotation (*p* = 0.14) for AD^63^ (Figures 4C and 4D). The enrichment of “later” PCs that explain less variance suggests that subtle single-cell states may still be genetically and biologically meaningful, although often discarded by cell type and many state-based analyses.^14^^,^^15^ Importantly, these states would have been excluded using a conventional “elbow plot” cutoff rather than the BIC cutoff we employed (Figure S5). The heritability analysis supported that using states computed from 20,000 variable genes, as opposed to 3,000 (which is often used in current common practices), did not compromise the robustness of the results; in fact, heritability enrichments were more significant and enriched when using 20,000 variable genes (Figure 4) compared to 3,000 (Figure S8).

Next, we conducted GSEA on ResidPCA-based states most enriched for AD heritability to map disease-based states to their respective biological pathways. The state most significantly enriched for AD heritability was PC15 (AD heritability enrichment: 2.0, FDR *p* value: 1.9 × 10^−8^) (Figures 4C–4E). The enriched pathways in PC15 highlight a recently identified mechanism in AD,^16^ spanning the neuron-oligodendrocyte-microglia axis (PC15 correlation with states identified by iterative PCA: excitatory neurons, *R*^*2*^ 0.21; oligodendrocytes, *R*^*2*^ 0.14; microglia, *R*^*2*^ 0.08. Using GSEA, we found that this state connects early-stage amyloid production in neurons and oligodendrocytes with later-stage immune response and microglial activation, driving AD progression (Table S2). The state-based annotation, enriched for AD heritability and incorporating the top 200 genes with the most positive loadings, included a genome-wide significant AD locus overlapping RASGEF1C (ranked 88/200), suggesting a role for this state in the microglial immune response^64^ (Table S2). The second most significant ResidPCA-based state for AD heritability, PC27 (AD heritability enrichment: 2.0, FDR *p* value: 3.6 × 10^−8^), relates to AD metabolic dysregulation, enriched in GSEA pathways related to disruptions in cholesterol and steroid hormone metabolism modulated through cytochrome p450^65^^,^^66^ (Table S2). The most enriched cell types in this state are excitatory neurons and oligodendrocytes (both *R*^*2*^ 0.12, although this did not exceed our predefined threshold *R*^*2*^ of 0.20 for cell-type specificity), which are significantly affected by cholesterol and retinol metabolites that influence neuronal plasticity and myelin protection.^67^^,^^68^^,^^69^^,^^70^^,^^71^^,^^72^ A cell-type-specific state identified by ResidPCA is PC44 (AD heritability enrichment: 1.8, FDR *p* value: 2.5 × 10^−5^), which is enriched in microglia (as indicated by GSEA enrichment, although insufficiently powered to reach significant correlation with iterative PCA microglial states, *R*^*2*^ 0.05) and enriched for AD heritability. This state contrasts with the previously mentioned states that span multiple cell types (Figure 4E; Table S2). It is associated with microglial activation modulated by CD8+ T cell signaling, highlighting the interplay between peripheral immune signaling and central nervous system-resident microglia in response to AD^73^ (Note S5).

To assess whether ResidPCA provides additional mechanistic insights compared to iterative PCA, we aggregated and quantified gene set enrichments across states for each method, rather than interpreting each state individually using GSEA. Overall, ResidPCA identified roughly twice as many significant GSEA enrichments in its RNA (6,726 enrichments)- and ATAC (6,340 enrichments)-based states compared to iterative PCA applications to each cell type (RNA: <3,146 enrichments, ATAC: <3,755 enrichments) (Figure 4F). GSEA enrichments identified by each PCA method were then grouped by pathway into broader categories, including inflammatory, brain-related, and metabolic pathways, where the same relationship was recovered (Figure 4G). In ResidPCA, inflammation-related pathways were the most enriched in RNA-related states (932 enrichments), followed by growth and proliferation response pathways (247 enrichments) and brain-enriched pathways (231 enrichments) (Figure 4G). Similarly, in ATAC-related states, inflammation was also the most enriched pathway (364 enrichments), followed by metabolism-related pathways (75 enrichments) and brain-related pathways (59 enrichments) (Figure 4G). To compare the overlap of enrichments between ResidPCA and iterative PCA applications to each cell type, we analyzed the overlapping and non-overlapping gene set enrichments identified by all iterative PCA applications versus ResidPCA (Figure 4H). We found that 56% and 55% of RNA and ATAC enrichments identified by ResidPCA, respectively, did not overlap with iterative PCA-identified enrichments (Figure 4H). Overall, iterative PCA, the method typically used to identify states within cell types in sequencing datasets, identified many fewer states and displayed a reduced number of gene set enrichments compared to ResidPCA.

In studies of AD, a key question is whether the pathways driving AD overlap with accelerated age-related processes or represent disease-specific mechanisms independent of general aging phenotypes.^74^^,^^75^ To address this, we refined our analysis of ResidPCA-based states to distinguish between pathways contributing to AD via aging-related processes and those functioning through disease-specific mechanisms. Using a combination of GSEA and heritability analysis, we evaluated whether the identified states were enriched for AD heritability, gene sets associated with aging, and/or gene sets associated with AD.

Our analysis identified 22 ResidPCA-based states enriched for AD heritability without significant enrichment for either aging or AD gene sets, suggesting that these states are driven by disease-specific pathways independent of aging phenotypes (Figure 4I). Conversely, PC6, identified by ResidPCA, showed enrichment for AD heritability (enrichment: 2.1, FDR *p* value: 2.5 × 10^−5^), aging gene sets, and AD gene sets (Figure 4I). This state is predominantly microglial (*R*^*2*^ = 0.23) and encompasses key AD-related pathways, including the TYROBP microglial signaling network, which involves the TREM2 receptor that binds amyloid-beta and activates microglial inflammatory responses (Table S2).^76^ Further analysis of the annotation within this state, enriched for AD heritability and comprising the bottom 200 genes with the most negative loadings in PC6, identified genome-wide significant markers for AD, including APP (ranked 195/200), a precursor to amyloid-beta plaques, and HERC1 (ranked 112/200), associated with protein degradation and inflammation. These findings suggest that PC6 represents a mechanistic intersection of microglial detection of APP- and AD-specific inflammatory signaling within the broader age-related pathway of inflammation.

In contrast, we identified 7 ResidPCA states with heritability enrichment and aging gene set enrichment but no AD gene set enrichment (Figure 4I). These states likely contribute to AD through the general acceleration of age-related processes, which are amplified in aging populations but are not exclusive to AD. These pathways represent broad aging mechanisms that predispose individuals to AD as well as other aging-related phenotypes. For example, PC3 is a state spanning 3 glial cell types—astrocytes (*R*^*2*^ = 0.38), microglia (*R*^2^ = 0.25), and oligodendrocytes (*R*^*2*^ = 0.35)—and reflects pathways characteristic of aging rather than AD-specific pathology. Enriched pathways in PC3 include reduced glial differentiation, impaired axon ensheathment (a key oligodendrocyte function), cholesterol biosynthesis, and protein refolding. These findings suggest that PC3 represents an interactive phenotype where general aging pathways contribute to AD risk without being directly specific to the disease (Note S5).

These findings highlight the distinction between states enriched for AD-specific pathways, such as PC6, which reflect mechanisms directly tied to AD pathology, including microglial signaling and amyloid-beta plaque detection, and those enriched for general aging pathways, such as PC3, which contribute to AD through indirect, age-related mechanisms. By distinguishing between these 2 enrichment profiles, this analysis advances the understanding of how distinct states drive AD and identifies opportunities for targeted interventions addressing both AD-specific mechanisms and aging-related vulnerabilities.

### Implementation of ResidPCA in an efficient computational software package

To facilitate broad adoption of our methodology, we have developed a computational pipeline named the ResidPCA Toolkit. This pipeline efficiently reproduces many of the analyses described in this paper, outperforming existing methods in both computational efficiency and resource usage. The ResidPCA Toolkit achieves this by (1) integrating a fast randomized SVD algorithm for PC calculation and (2) pre-computing a single covariate matrix inversion for expression residuals, avoiding repeated inversions for each target gene.

**Figure 5:**
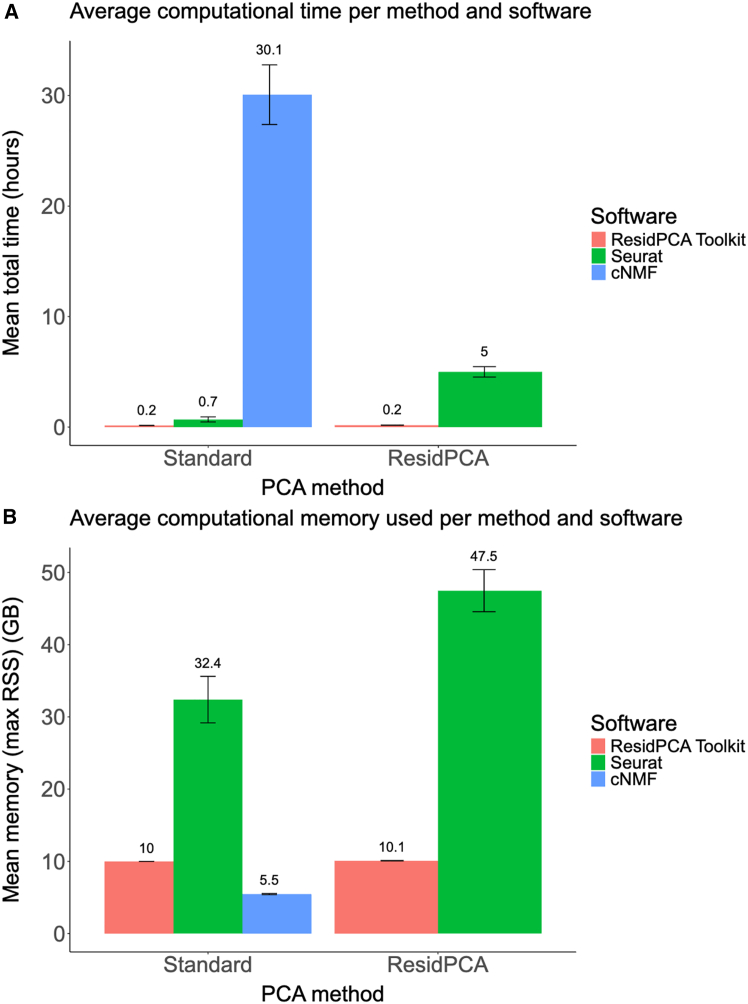
The ResidPCA Toolkit enables efficient and scalable PCA-based state discovery Average computational (A) time and (B) memory usage across methods and software. Performance was evaluated using count matrices of varying dimensionalities (i.e., differing numbers of cells and genes). Performance was averaged across count matrix input sizes. (Left) The average time (A) and memory (B) required by the ResidPCA Toolkit and Seurat to run standard PCA and by the cNMF package to run cNMF. (Right) The corresponding averages for ResidPCA, as implemented in the ResidPCA Toolkit and Seurat. ResidPCA identifies disease-relevant cell states by modeling heterogeneity across cell types. Applied to mouse visual cortex and AD datasets, ResidPCA outperformed existing methods and uncovered chromatin and transcriptional states enriched for heritability, offering mechanistic insights into neurodegeneration. Error bars indicate one standard error of the mean.

We evaluated the performance of the ResidPCA Toolkit by comparing it with state-of-the-art methods: standard PCA implemented with Seurat^28^ and cNMF^12^ implemented with the cNMF package. Our comparison focused on evaluating the maximum memory usage (maximum resident set size [RSS]) and central processing unit (CPU) time across a range of simulated count matrix sizes, which varied by both the number of genes and the number of cells in the matrices. Despite handling a more complex task, ResidPCA implemented with the ResidPCA Toolkit required less CPU time than standard PCA implemented with Seurat and cNMF implemented with the cNMF package (Figure 5A). Specifically, the average CPU time for ResidPCA using the ResidPCA Toolkit was 0.17 h (standard error [SE] 0.010). In comparison, standard PCA implemented with Seurat took 0.69 h (SE 0.22) and cNMF using the cNMF package required 30.08 h (SE 2.70) (Figure 5A). For a count matrix containing 100,000 cells and 5,000 genes, standard PCA with Seurat and cNMF with the cNMF package required 9 and 157 times more CPU time, respectively, than ResidPCA using the ResidPCA Toolkit (Figure S9). In terms of memory usage, ResidPCA using the ResidPCA Toolkit consumed less memory, with an average maximum RSS of 10.08 GB (SE 0.029) compared with standard PCA using Seurat, which used 32.39 GB (SE 3.22) and ResidPCA using Seurat, which required 47.47 GB (SE 2.91). cNMF using the cNMF package required slightly less memory, 5.47 GB (SE 0.087) (Figure 5B). The key memory difference between cNMF and the ResidPCA Toolkit is that the ResidPCA toolkit saves the outputted embeddings and loadings into memory so that they can be interactively used; however, the memory differences are not huge. When comparing the ResidPCA Toolkit with Seurat for performing ResidPCA (an identical task), the ResidPCA Toolkit required less CPU time and memory (Figures 5A and 5B), with its performance scaling linearly with the number of genes (Figure S9; Note S6).

## Discussion

Our study introduces ResidPCA, a method for identifying cellular states in single-cell sequencing data by conditioning out cell-type heterogeneity. By analyzing all cells simultaneously, ResidPCA enables the discovery of cell states that both span multiple cell types and are enriched within a single cell type. In contrast, iterative PCA^14^ and other well-established methods^15^^,^^16^^,^^19^^,^^20^^,^^21^ primarily focus on identifying states within a single predefined cell type. Through extensive simulations and analysis of experimental datasets, ResidPCA excels in identifying a wide spectrum of states ranging from rare to abundant, with higher accuracy and sensitivity compared to well-established methods. Notably, ResidPCA was able to identify rare states and those with high cell-type heterogeneity in simulation where other methods failed. In real data, ResidPCA greatly outperformed NMF-based methods in detecting light-induced states, particularly in endothelial and smooth muscle and excitatory neurons, the cell types most impacted by light exposure.^31^ Separately, ATAC-based states produced more enrichment than RNA-based states, consistent with previous analyses,^3^^,^^60^^,^^63^ and ResidPCA consistently revealed more states enriched for heritability than states inferred by other methods or conventional cell-type annotations.

The extent to which single-cell measurements can provide insights into disease heritability and mechanisms has been an open question.^3^^,^^5^ In our analyses, state annotations consistently showed higher disease heritability enrichments than cell-type annotations, making the identification of cell states beneficial for several reasons.^3^^,^^5^^,^^77^ First, even though disease heritability is typically enriched in genes expressed in relevant cell types or tissues, most of the absolute disease heritability still tends to reside in genes that are broadly expressed across tissues,^78^ implicating state-like processes that span many cell types. Second, attributing disease heritability solely to cell types may only tag the disease-related state prevalent in the predominant cell type associated with the disease. However, some cells within this enriched cell type may be “healthy cells” not actively contributing to the disease process. The inclusion of genes from these healthy cells in heritability analyses may weaken the observed heritability enrichment. Finally, cell types can often provide only a high-level localization of disease heritability—for example, showing that the heritability of neurological conditions is enriched in neurons.^3^ By using states as annotations for heritability enrichment, we can pinpoint more specific biological functions driving disease, rather than merely tagging the cell types capable of assuming or not assuming a given disease-related state. This knowledge is crucial for the development of targeted therapeutics that address the specific cellular states driving disease progression, rather than targeting broad cell types.

We make several recommendations for inference of cell states that differ from conventional strategies. First, even after dimensionality-reduction methods are applied, inferred states are often visualized using UMAP for face validity and interpretation. However, our simulations showed that 2 UMAP dimensions often reflect cell-state relationships poorly, consistent with prior work,^41^ and we caution against interpreting states visually from such a low-dimensional representation.

Second, while prior methods typically restrict single-cell data to a small number of dimensions for downstream clustering (e.g., UMAP for Seurat), we found that single-cell datasets contain greater dimensionality in the data than is typically considered.^10^^,^^14^^,^^23^ Using the BIC to determine the number of significant states identified by a given method resulted in many more significant states than using heuristics such as the elbow cutoff. This advantage is particularly pronounced in ResidPCA, which applies BIC to the full dataset. In contrast, iterative PCA is constrained by reduced sample size, limiting the number of components that can be reliably retained and thereby reducing power to detect significant states. By comparison, NMF-based approaches rely on heuristic rules for dimensionality selection that can lead to unstable results: small changes in the chosen number of components often cause large and unpredictable drops in accuracy, especially in large-scale single-cell datasets with diverse cell types (Figure S10). Finally, hard clustering, a widely used method,^10^^,^^14^^,^^23^ imposes strong assumptions by restricting each cell to a single state and requiring states to be exclusive to 1 cell type, assumptions that are inconsistent with established biology.^79^ Our observations show that many states can be shared across cell types and that individual cells can express multiple states, highlighting the limitations of hard clustering and providing insights into how different cell types collaborate to drive disease.

Third, single-cell pipelines often restrict analyses to a subset of highly variable genes to capture the largest sources of biological variation. While these genees effectively represent expression patterns driven by cell type, cell states--being rarer and contributing less overall variation--are more difficult to detect. Consequently, the ability to identify cell states can be affected by the number of genes included in the analysis. Our analyses demonstrate that incorporating additional non-highly variable genes can improve the sensitivity of state detection in some settings but not in others. Defining the precise number or weighting of genes to use for dimensionality reduction in a given experiment thus remains an open question, although we recommend exploratory analyses with a large number of genes.

While ResidPCA is generally more accurate and sensitive to detecting cell states compared to well-established methods, there are some limitations. First, cell state(s) highly correlated with cell type may be poorly detected due to the removal of cell-type effects. While we believe the residual cell-state component may be particularly informative regarding biological mechanisms that are independent of cell type, this approach may miss certain cell states or provide a distorted representation of their activity. Second, in modeling cell-type expression, discrete cell-type labels were used; however, continuous or probabilistic cell-type assignments can be incorporated that may improve cell-type expression estimation, especially for differentiating or difficult-to-classify cell types. Relatedly, when marker-based classification is poor, some cell types may be obscured. In such cases, cell-type detection may suffer; however, cell-state detection will remain comparable to standard PCA, provided the cell state is not highly correlated with the regressed-out cell type. Third, benchmarking methods for cell-state inference is hindered by the general lack of well-established ground truth cell states. We sought to overcome this limitation by evaluating realistic simulations, experimental data under light stimulation, and large-scale data at steady state. However, more refined techniques and standardized datasets of cell states are needed, as have been collected for cell types through single-cell atlases.^2^ Fourth, ResidPCA does not account for the inherent sparsity in single-cell data, which is driven by technical variations such as stochasticity in cell lysis, reverse transcription efficiency, and molecular sampling during sequencing,^80^ as well as biological variation such as transcriptional bursting.^81^ While negative binomial or gamma-Poisson models are generally a good fit for these types of data, zero-inflated negative binomial models can sometimes capture sparsity better.^25^ This indicates the potential benefit of incorporating a sparsity prior into simulation and state detection.

Alternative approaches for connecting single-cell data to heritability include methods such as scDRS^82^ and SCAVENGE,^83^ which project GWAS data into the single-cell space. These methods aim to map genetic associations identified in GWAS to specific cell types and states, providing insights into the cellular context of genetic variants. In contrast, our approach projects single-cell annotations onto GWAS heritability, focusing on how single-cell data can inform the genetic architecture of diseases. This reverse mapping is effective when there is rich structure in the single-cell data, allowing for precise identification of disease-relevant cellular states. However, GWAS-based methods may be more advantageous when many causal genes are accurately implicated by GWAS, facilitating the identification of relevant cell types and states. Both approaches offer valuable perspectives, and their effectiveness depends on the complexity and resolution of the underlying data. In parallel to these association-based strategies, method development has also focused on improving how single-cell data are processed and compared. For example, Lee and Han^84^ proposed a fast approach for batch-adjusted PCA in single-cell data, although it does not currently model continuous cell types or covariates—an area for future work. Another complementary direction is to harmonize a specific cell type across multiple cohorts or conditions, enabling traditional statistical methods to be applied within a more controlled context (e.g., Kotliar et al.^85^). While our approach focuses on leveraging all cells simultaneously, this single-cell-type-focused strategy may be better suited when the relevant population is clearly defined and consistent across datasets.

In terms of future work, the cell states inferred by ResidPCA may also serve as informative annotations for the identification of single-cell quantitative trait loci (QTLs) and QTL heritability.^10^^,^^14^ This could enable the detection of variants that regulate specific cellular programs rather than broad cell types, improve fine-mapping by linking regulatory variants to precise states, and integrate naturally with multi-omic data to capture additional layers of regulation.

ResidPCA offers a powerful tool for identifying biologically relevant states and their associated cell types, thereby advancing our understanding of disease mechanisms. Moreover, its ability to leverage all cells enhances statistical power, making it especially valuable in scenarios where the subset of relevant cell types is unknown, rare states are present, or sample size is low. We present a comprehensive computational pipeline and software implementation of ResidPCA, providing researchers with a powerful, user-friendly tool to compare PCA methods, characterize cell states, and investigate their roles across cell types and complex diseases.

## Resource availability

### Lead contact

Requests for further information and resources should be directed to and will be fulfilled by the lead contact, Shaye Carver (scarver@g.harvard.edu).

### Materials availability

This study did not generate new unique reagents.

### Data and code availability

All data used in this study are publicly available. Multimodal datasets, cell-type proportion estimates, and fitted parameters were obtained from Morabito et al.^15^ (Synapse: syn22795973). The light exposure expression dataset was obtained from Hrvatin et al.^31^ (GEO: GSE102827). The ResidPCA Toolkit, together with Shell and Python script tutorials for running the package and code for simulation analyses, is available at https://github.com/carversh/residPCA. Analyses were performed using R (version 4.1.0) and Python (version 3.8.18). There are no restrictions on the availability of either the data or the code.

## Acknowledgments

We extend our sincere gratitude to B. Tolooshams, A. Lin, D. Ba, M. Theodosis, and D. Yao for their valuable insights during discussions on computational methods and single-cell data. We also thank Y. Ding for their creative input in naming the method. S.C. acknowledges support from the 10.13039/100023581National Science Foundation Graduate Research Fellowship Program. This work was supported by 10.13039/100000002NIH grants R01MH115676, R01HD114855, R01HG012133, and R01MH12525.

## Author contributions

A.G. and S.C. conceptualized the study, designed the methodology, and led the statistical analysis. All authors assisted in computational analyses, provided revisions to the manuscript and contributed to the final draft. All authors read and approved the final manuscript.

## Declaration of interests

S.G. is an employee of GSK.

## Declaration of generative AI and AI-assisted technologies in the writing process

During the preparation of this work, the authors used ChatGPT to enhance the readability and language of the manuscript. After utilizing this tool/service, the authors thoroughly reviewed and edited the content as necessary and take full responsibility for the final content of the published article. The use of generative AI and AI-assisted technologies was solely intended to improve the clarity and presentation of the manuscript.

## Web resources

Code availability: https://github.com/carversh/residPCA

## Supplemental information

Document S1. Figures S1–S10, Tables S1 and S2, and Notes S1–S6

Document S2. Article plus supplemental information
